# Thirty Years of Hide-and-Seek: Capturing Abundant but Elusive M^III^@*C*_3*v*_(8)-C_82_ Isomer, and the Study of Magnetic Anisotropy Induced in Dy^3+^ Ion by the Fullerene π-Ligand

**DOI:** 10.1021/jacs.4c10050

**Published:** 2024-09-02

**Authors:** Wei Yang, Matheus Felipe de Souza Barbosa, Alexey Alfonsov, Marco Rosenkranz, Noel Israel, Bernd Büchner, Stanislav M. Avdoshenko, Fupin Liu, Alexey A. Popov

**Affiliations:** a Leibniz Institute for Solid State and Materials Research (IFW Dresden), Helmholtzstrasse 20, 01069 Dresden, Germany; b Jiangsu Key Laboratory of New Power Batteries, School of Chemistry and Materials Science, Nanjing Normal University, Nanjing 210023 China

## Abstract

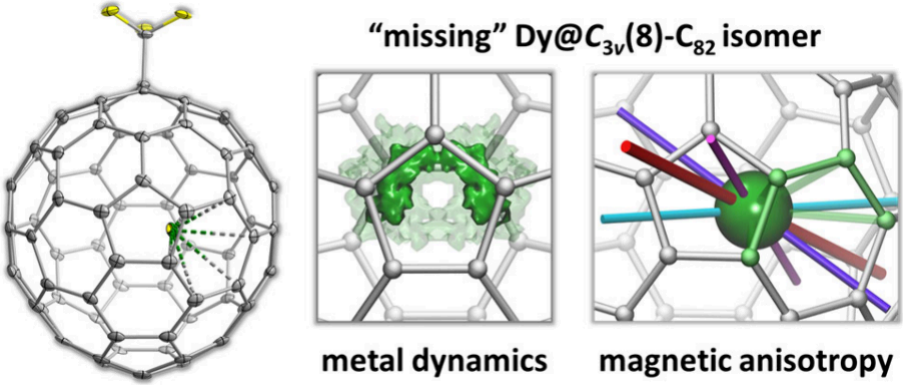

Our knowledge about endohedral metallofullerenes (EMFs) is restricted to the structures with sufficient kinetic stability to be extracted from the arc-discharge soot and processed by chromatographic and structural techniques. For the most abundant rare-earth monometallofullerene M^III^@C_82_, experimental studies repeatedly demonstrated *C*_2*v*_(9) and *C*_*s*_(6) carbon cage isomers, while computations predicted equal stability of the “missing” *C*_3*v*_(8) isomer. Here we report that this isomer is indeed formed but has not been recovered from soot using standard protocols. Using a combination of redox extraction and subsequent benzylation and trifluoromethylation with single-crystal XRD analysis of CF_3_ adduct, we prove that Dy@*C*_3*v*_(8)-C_82_ is one of the most abundantly produced metallofullerenes, which was not identified in earlier studies because of the low kinetic stability. Further, using the Dy@*C*_3*v*_(8)-C_82_(CF_3_) and Dy@*C*_3*v*_(8)-C_82_(CH_2_Ph) monoadducts for the case study, we analyzed the role of metal-fullerene bonding on the single-ion magnetic anisotropy of Dy in EMFs. The multitechnique approach, combining *ab initio* calculations, EPR spectroscopy, and SQUID magnetometry, demonstrated that coordination of the Dy ion to the fullerene cage induces moderate, nonaxial, and very fluid magnetic anisotropy, which strongly varies with small alterations in the Dy-fullerene coordination geometry. As a result, Dy@*C*_3*v*_(8)-C_82_(CH_2_Ph) is a weak field-induced single-molecule magnet (SMM), whose signatures of magnetic relaxation are detectable only below 3 K. Our results demonstrate that metal-cage interactions should have a detrimental effect on the SMM performance of EMFs. At the same time, the strong variability of the magnetic anisotropy with metal position suggests tunability and offers strategies for future progress.

## Introduction

**Figure 1:**
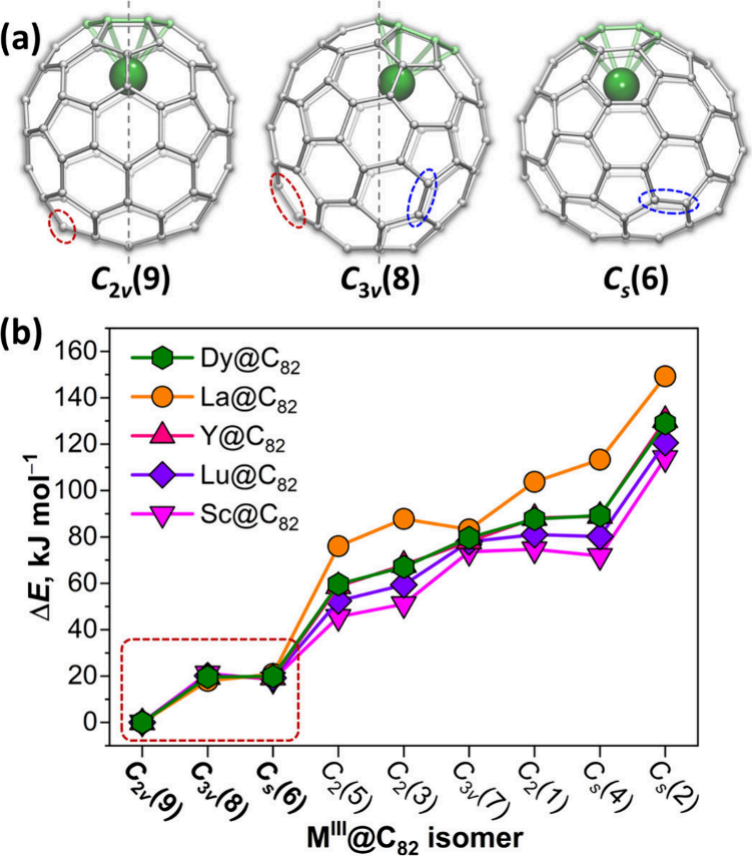
(a) Molecular structures of M^III^@C_82_ metallofullerenes with *C*_2*v*_(9), *C*_3*v*_(8), and *C*_*s*_(6) cage isomers; vertical dashed gray lines denote symmetry axes, dashed ovals mark C–C bonds, which undergo the Stone-Wales transformation (90° pseudorotation) on going from *C*_2*v*_(9) to *C*_3*v*_(8) (red) or from *C*_*s*_(6) to *C*_3*v*_(8) isomers (blue). (b) DFT (PBE) relative energies of nine IPR isomers of M^III^@C_82_ computed for representative rare-earth metals with different ionic radii (the points for Y are not well seen because they nearly coincide with Dy); the three most stable isomers, whose structures are shown in (a), are highlighted by the dashed red rectangle.

Monometallofullerenes (mono-EMF) M^III^@C_82_, where M^III^ is a trivalent rare earth metal, are arguably the most studied type of endohedral metallofullerenes (EMFs), only rivaled in this role by nitride clusterfullerenes M_3_N@C_80_.^1−4^ Already the first reports on the synthesis of EMFs in the early 1990s showed M@C_82_ to be the most abundantly produced EMF species,^5−7^ which facilitated their accumulation for further chemical and physical studies. In 1994, Yamamoto et al. isolated the minor isomer of La@C_82_, proving that M^III^@C_82_ actually consists of two isomers.^8^ Based on synchrotron powder X-ray diffraction (XRD) with maximum entropy method refinement,^9−11^^13^C NMR measurements of M@C_82_ anions,^12−17^ and then on numerous single-crystal X-ray diffraction (SC-XRD) studies,^18−28^ the structures of the major and minor isomers of M^III^@C_82_ were assigned to *C*_2*v*_(9) and *C*_*s*_(6) cages (Figure 1a), respectively. For years, the isomeric composition of M^III^@C_82_ comprising these two structures has been common knowledge in the fullerene field.

Isomerism of fullerenes can be full of surprises because we are able to process and structurally characterize only those of them which can be extracted from the arc-discharge soot. It means that some fullerene structures, which are formed in the synthesis, may pass under the radar and remain unknown if they are not soluble in common solvents. The reason for the poor solubility is a low kinetic stability, which implies that “insoluble” fullerenes polymerize or react with other carbonaceous species present in the soot. In such situations, changing the workup protocol can make them available. Stabilization and solubilization of kinetically unstable fullerenes is usually accomplished by an electron transfer, chemical functionalization, or a combination of both.^29,30^ The prominent examples include empty fullerene *D*_3*h*_-C_74_, which remained “missing” until its formation was revealed by electrochemical reduction and dissolution in the anionic form.^31^ Later, radical trifluoromethylation of the mixture of C_74_ and other insoluble fullerenes allowed their solubilization, separation, and structural characterization in the form of C_2*n*_(CF_3_)_12_ derivatives and provided the first evidence for several hitherto unknown empty fullerenes, including *T*_*d*_(2)-C_76_, *D*_3*h*_(5)-C_78_, *C*_2*v*_(5)-C_80_, and *C*_2_(5)-C_82_.^32,33^ Modification of this method by Shinohara et al. for *in situ* trifluoromethylation during EMF synthesis helped to capture elusive M@C_60_ and M@C_70_ (M = Y, La, Gd) in the form of their CF_3_ adducts.^34,35^ Dimetallofullerene (di-EMF) M_2_@C_80_, with M being a trivalent rare earth metal other than La, Ce or Pr, is another example of elusive EMF. M_2_@C_80_ was often detected by mass spectrometry, but could not be obtained in an appreciable amount for the structure elucidation until extraction of EMF soots with boiling dimethylformamide followed by chemical functionalization afforded the isolation and structural characterization of M_2_@C_80_(CH_2_Ph) and M_2_@C_80_(CF_3_) derivatives (M = Y, Nd, Gd, Tb, Dy, Ho, Er) in our group.^36−39^ Akasaka et al. found that when halobenzenes are used as solvents in the EMF extraction, they also produce aryl radicals, which tend to attach to insoluble EMFs and form their soluble adducts.^40,41^ Several “missing” mono-EMFs were captured by this method,^42−45^ including the third isomer of La@C_82_ with the *C*_3*v*_(7)-C_82_ cage.^46^ To the best of our knowledge, aside from this single report on the *C*_3*v*_(7) isomer, there has been no experimental evidence for the isomers of M^III^@C_82_ other than those of *C*_2*v*_(9) and *C*_*s*_(6).

There is broad consensus that the isomeric composition of fullerenes formed in the arc-discharge synthesis is at least partially controlled by thermodynamic factors. That means, experimentally available structures are usually among the most stable ones predicted theoretically.^47,48^ Although some isomers with high relative energies are also occasionally isolated, they are believed to be links or traps on the rearrangement pathways between more stable structures.^49−52^ Thus, it is almost certain that the most stable isomers predicted by DFT will be experimentally available, although the opposite is not necessarily true and the high relative energy of a given isomer does not guarantee its absence in the products of synthesis. Computational study of M^III^@C_82_ (M = Y, La) isomers reported by Kobayashi and Nagase back in 1998 showed that in accordance with its high experimental abundance, *C*_2*v*_(9)-C_82_ was the most stable isomer of M^III^@C_82_.^53^ Next in the stability order was the *C*_3*v*_(8)-C_82_ isomer with the relative energy of 18–20 kJ mol^–1^, followed by similarly stable *C*_*s*_(6)-C_82_. Several studies of the relative energies and isomeric composition of M@C_82_ (M = Y, La, Ce, Pr, Gd, Er, Lu) performed later by Slanina et al. with the use of DFT and statistical thermodynamics repeatedly showed comparable stability of *C*_*s*_(6) and *C*_3*v*_(8) isomers and considerable equilibrium concentration of the latter.^54−60^ Our DFT calculations for M^III^@C_82_ with rare-earth metals of different ionic radius (M = Y, La, Dy, Lu, Sc) show similar results (Figure 1b, S1, Table S1). Thus, while the predicted stability of *C*_2*v*_(9) and *C*_*s*_(6) isomers agrees with their experimental availability, the lack of experimental reports on M^III^@*C*_3*v*_(8)-C_82_ is surprising. At the same time, on a par with *C*_*s*_(6)-C_82_, the *C*_3*v*_(8)-C_82_ cage is one of the most abundant isomers for metallofullerenes with the 4-fold electron transfer,^61^ including a variety of dimetallofullerenes M_2_@*C*_3*v*_(8)-C_82_ (M = Sc,^62^ Y,^63^ Dy,^64^ Er,^65^ Lu^66,67^), clusterfullerenes M_2_C_2_@*C*_3*v*_(8)-C_82_ (M = Sc,^68^ Y^69^), M_2_O@*C*_3*v*_(8)-C_82_ (M = Sc,^70^ Dy^71^), M_2_S@*C*_3*v*_(8)-C_82_ (M = Sc,^72,73^ Er,^66^ Dy^74,75^), and actinide mono-EMF Th^IV^@*C*_3*v*_(8)-C_82_.^76^ Recently, it was also determined in azafullerene La@C_81_N,^77^ in which the C_81_N^3–^ cage is isoelectronic to C_82_^4–^. These multiple examples demonstrate that there are no fundamental reasons preventing EMFs with the *C*_3*v*_(8)-C_82_ cage from being formed in the arc-discharge process. Furthermore, the structure of the *C*_3*v*_(8) isomer is related to the *C*_2*v*_(9) and *C*_*s*_(6) isomers by a single Stone-Wales transformation, showing a close similarity between the three cages (Figure 1a). However, neither standard nor modified workup protocols could recognize the formation of rare-earth mono-EMFs with the *C*_3*v*_(8)-C_82_ cage so far.

Here we demonstrate that although the M^III^@*C*_3*v*_(8)-C_82_ isomer avoided being discovered for more than 30 years of EMF research, it is not only formed in the arc-discharge synthesis, but also one of the most abundantly produced EMFs second to only M^III^@*C*_2*v*_(9)-C_82_. It took a combination of redox extraction and chemical functionalization to obtain CH_2_Ph and CF_3_ adducts of Dy@*C*_3*v*_(8)-C_82_ and unambiguously determine their molecular structure by single-crystal X-ray diffraction. Profiting from the well-defined molecular structure of the Dy@C_82_ adducts, we also address the question of a dual role of the fullerene cage as a π-ligand and an electron acceptor in determining the magnetic anisotropy of an endohedral Dy^3+^ ion.

## Results and Discussion

### Synthesis and Molecular Structure of Dy@*C*_3*v*_(8)-C_82_ Adducts

#### Redox Extraction and Functionalization of Dy-EMFs

In 2017, aiming at the stabilization of dimetallofullerenes with a single-electron metal–metal bond, we developed a combination of redox extraction of EMFs from the arc-discharge soot by dimethylformamide (DMF) with thermal benzylation of extracted EMF anions.^38,39^ The high efficiency of DMF in extracting metallofullerenes was discovered in the late 1990s.^78−80^ The key element of the process is the reduction of EMFs to anionic form,^81^ presumably by amines formed during the thermal decomposition of DMF. Reduction increases kinetic stability of many EMFs with open-shell electronic structure (such as mono and di-EMFs). At the same time, EMF anions are readily soluble in polar DMF (unlike noncharged fullerenes, whose solubility in DMF is low). We then found that under controlled heating, EMF anions in DMF react with benzyl bromide by substituting the bromide ion and forming neutral air-stable benzyl monoadducts soluble in toluene.^39^ Reaction is not selective toward any specific fullerene in a way that all EMF anions equally end up as EMF(CH_2_Ph) monoadducts. Since the regioselectivity of benzylation is also low in these conditions and one EMF can produce several regioisomers of EMF(CH_2_Ph), the composition of the product mixture is quite complex. However, it is amenable to chromatographic separation, which allowed isolation of target Dy_2_@C_80_(CH_2_Ph), reported in ref (39), as well as some other EMF(CH_2_Ph) adducts, including Dy@C_80_(CH_2_Ph) and several isomers of Dy@C_82_(CH_2_Ph) (Figure S2). Analogous EMF(CH_2_Ph) mixtures were also obtained for Y, Gd, Tb, Ho, and Er.^38,39^ Benzyl derivatives do not crystallize easily, and when they do they often form crystals of poor quality, which prevented the structure elucidation of mono-EMF adducts at that time. Recently, using the same extraction/benzylation protocol and utilizing cocrystallization with decapyrrylcorannulene for SC-XRD analysis, Yang et al. assigned molecular structure of Dy@C_80_(CH_2_Ph) to the *C*_2*v*_(5)-C_80_ isomer.^82^

Looking for a more convenient reaction for functionalization of EMF anions, we developed electrophilic trifluoromethylation by 2,8-difluoro-5-(trifluoromethyl)-5H-dibenzo[b,d]thiophen-5-ium trifluoromethanesulfonate,^83^ also known as Umemoto reagent II. Reaction proceeds almost instantaneously at room temperature and is formally described as an addition of the CF_3_^+^ cation to EMF anions with formation of EMF(CF_3_) adducts, which can be then dissolved in toluene and separated by HPLC. The real mechanism is more complicated, including a formation of CF_3_ polyadducts at later stages, which requires careful control of the reagent ratio. Under the optimized ratio, the reaction is remarkably selective toward M_2_@*I*_*h*_-C_80_, giving M_2_@*I*_*h*_-C_80_(CF_3_) as the main toluene-soluble reaction product. First developed for Tb-EMFs and Y-EMFs,^37^ it worked equally well for Nd-EMFs,^36^ and in this work for Dy-EMFs. In addition to the main product, Dy_2_@*I*_*h*_-C_80_(CF_3_), HPLC separation also afforded isolation of several isomers of Dy@C_82_(CF_3_) as minor products (Figure S3).

#### Molecular Structures of Dy@*C*_3*v*_(8)-C_82_(CF_3_) and Dy@*C*_3*v*_(8)-C_82_(CH_2_Ph)

**Figure 2:**
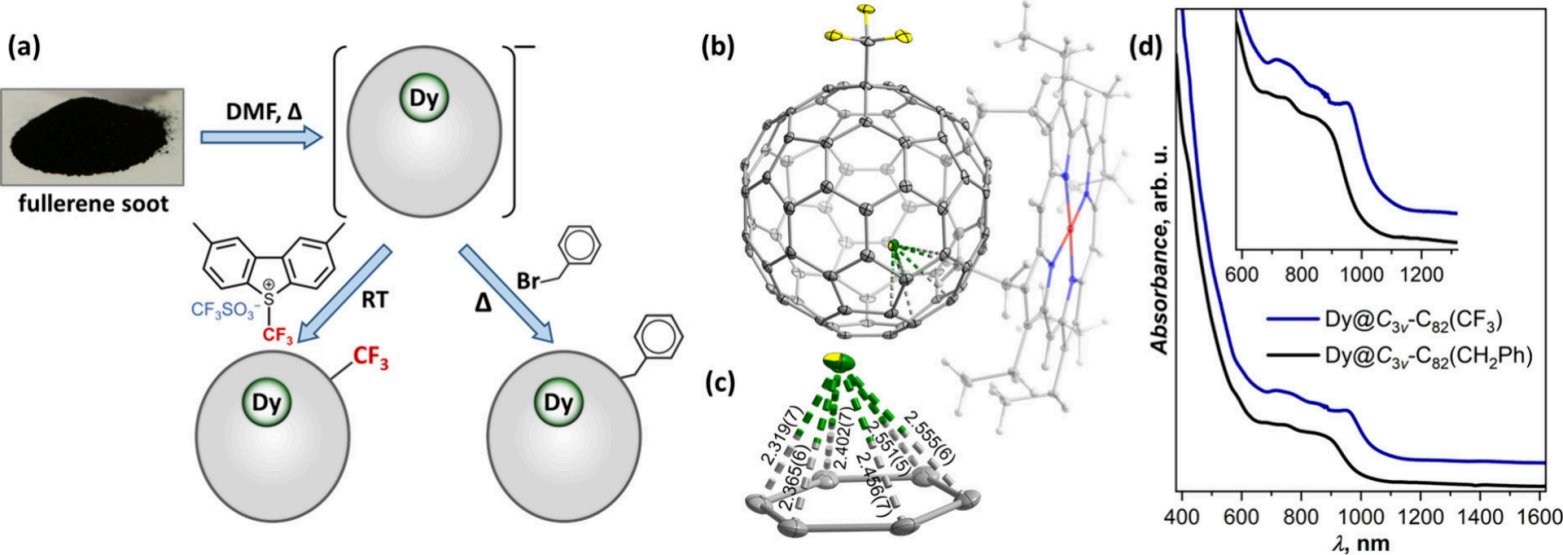
(a) Extraction and functionalization of Dy-EMFs with CF_3_ and CH_2_Ph groups. (b) Single-crystal structure of Dy@*C*_3v_(8)-C_82_(CF_3_) with coordinated NiOEP (shown semitransparent); fullerene molecule is viewed along the *C*_3_ symmetry axis of the *C*_3*v*_(8)-C_82_ cage; solvent molecules are omitted for clarity, F – yellow, C – gray, Dy – green, Ni – red, and N – blue. (c) Dy-coordinated hexagon with Dy–C distances (in Å). (d) Vis–NIR absorption spectra of isostructural Dy@*C*_3*v*_(8)-C_82_(CF_3_) and Dy@*C*_3*v*_(8)-C_82_(CH_2_Ph).

SC-XRD analysis of one of the Dy@C_82_(CF_3_) isomers, cocrystallized with Ni octaethylporphyrin (NiOEP), provided well-ordered structure shown in Figure 2b,c and Figure S5–S7. The fullerene cage is unambiguously determined as *C*_3*v*_(8)-C_82_, giving the first experimental evidence for this isomer of M^III^@C_82_. CF_3_ group is attached to the [5,6,6] carbon on the symmetry plane of the fullerene cage. Dy atom is fully ordered and is coordinated to a hexagon in a η^6^-fashion, with Dy–C bonds ranging from 2.319(7) to 2.555(6) Å (Figure 2c, S6).

To identify the counterpart of Dy@*C*_3*v*_(8)-C_82_(CF_3_) among Dy@C_82_(CH_2_Ph) adducts, we employed UV–vis–NIR absorption spectroscopy. Absorption spectra of fullerene derivatives are determined by the π–π* excitations of the fullerene and are very sensitive to the π-system topology but almost insensitive to the nature of the addends. Indeed, we found that the absorption spectrum of one Dy@C_82_(CH_2_Ph) isomer showed a very close similarity to the spectrum of Dy@*C*_3*v*_(8)-C_82_(CF_3_) (Figure 2d), whereas the spectra of other isomers were quite dissimilar. As we discuss in more detail below, Dy@*C*_3*v*_(8)-C_82_(CH_2_Ph) appeared to be one of the main fractions, suggesting a high abundance of Dy@*C*_3*v*_(8)-C_82_. The shift of the lowest-energy band from 890 nm for CH_2_Ph to 955 nm for CF_3_ is similar to that observed for Tb_2_@*I*_*h*_-C_80_(CH_2_Ph) and Tb_2_@*I*_*h*_-C_80_(CF_3_) derivatives in ref (37) and points to the electron-withdrawing action of CF_3_ group, which affects the LUMO energy stronger than the HOMO.

**Figure 3:**
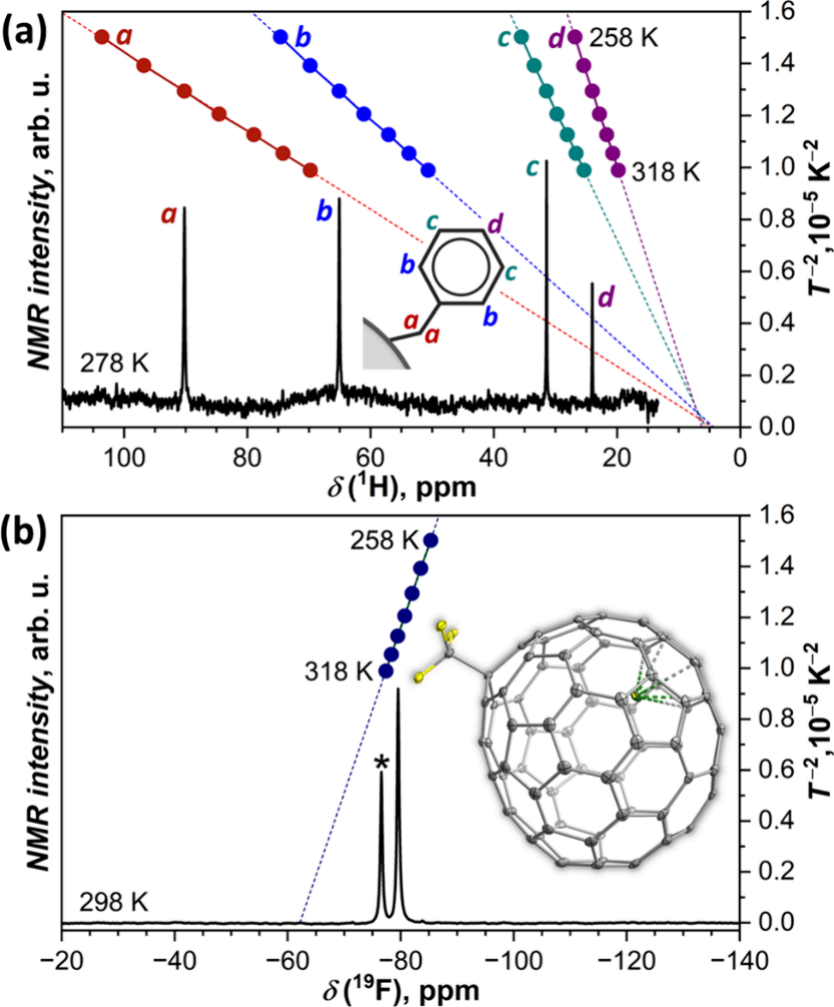
(a) ^1^H NMR spectrum of Dy@C_82_(CH_2_Ph) measured at 278 K, and chemical shifts of protons measured in the 258–318 K temperature range with the step of 10 K (dots); linear fits of temperature dependences in δ−*T*^–2^ coordinates and extrapolation to *T*^–2^ = 0 are shown with dashed lines. (b) ^19^F NMR spectrum of Dy@C_82_(CF_3_) measured at 298 K and chemical shift of the CF_3_ group measured in the 258–318 K temperature range with the step of 10 K (dots); linear fit of the temperature dependence in δ−*T*^–2^ coordinates and extrapolation to *T*^–2^ = 0 is shown with dashed line. The asterisk in (b) marks the signal of a CF_3_COOH solution in D_2_O, which was placed in the coaxial tube and used as the internal standard (δ = −76.55 ppm). The inset in (b) shows a different orientation of the Dy@C_82_(CF_3_) molecule than in Figure 2b

^19^F NMR spectrum of Dy@*C*_3*v*_(8)-C_82_(CF_3_) showed only one line (Figure 3, S8), consistent with the fast rotation of the CF_3_ group and with the high compositional and isomeric purity of the sample. Position of the signal varied from −85.33 ppm at 258 K to −77.41 ppm at 318 K, which is the expected behavior for a paramagnetic compound. The paramagnetic shift in Dy@C_82_(CF_3_) is similar to that in Nd_2_@C_80_(CF_3_)^37^ but much weaker than in Tb_2_@C_80_(CF_3_),^36^ which showed the variation of the signal from −286 ppm at 278 K to −222 ppm at 318 K. For comparison, chemical shifts of noncrowded CF_3_ groups in diamagnetic EMF derivatives span the range of −68 to −80 ppm.^84,85^

Benzyl group of Dy@C_82_(CH_2_Ph) gave four ^1^H NMR peaks in the range of 10–100 ppm (Figure 3, S9). The fact that only one peak is observed for methylene protons (labeled ***a*** in Figure 3a) shows that the benzyl group is attached to a carbon on a symmetry plane of the cage, in line with the SC-XRD structure of Dy@C_82_(CF_3_). The paramagnetic effect of Dy^3+^ in Dy@C_82_(CH_2_Ph) is more pronounced than that in Dy@C_82_(CF_3_) as follows from the range of the chemical shifts as well as from the stronger temperature variation. We will discuss paramagnetic NMR further below in the analysis of the magnetic anisotropy of Dy^3+^.

#### How Abundant is Dy@*C*_3*v*_(8)-C_82_?

It is hard to determine the real isomeric composition of as-synthesized EMF mixtures because of the different extraction behavior. Case in point is M^III^@*C*_3*v*_(8)-C_82_, which has not been even considered in earlier works. Since functionalization stabilizes kinetically unstable EMFs and makes them soluble, analysis of the mixture of derivatives seems more reliable under the condition that the reaction proceeds equally for all EMFs. From this point of view, electrophilic trifluoromethylation is not suitable for the analysis owing to its selectivity toward M_2_@*I*_*h*_-C_80_ and possible formation of multiadducts. Thermal benzylation, on the other hand, suits this goal very well.

Based on HPLC peak areas, we can estimate that the mixture of Dy-EMF(CH_2_Ph) adducts obtained after benzylation of the DMF fullerene extract contains 13% Dy@*C*_3*v*_(8)-C_82_(CH_2_Ph), 8% Dy@*C*_2*v*_(5)-C_80_(CH_2_Ph), 6–7% Dy_2_@*I*_*h*_(7)-C_80_(CH_2_Ph) and Dy_2_@*D*_5*h*_(6)-C_80_(CH_2_Ph) each, and the remaining 66% are mainly different isomers of Dy@C_82_(CH_2_Ph) and some low-abundant Dy-EMFs. If we assume that the mixture of Dy-EMFs before benzylation has the same composition, then Dy@C_82_ isomers should constitute around 70% of all Dy-EMFs in the DMF extract, and at least 18% of that amount has to be Dy@*C*_3*v*_(8)-C_82_. The actual content of *C*_3*v*_(8) is likely to be higher since molecular structures of all Dy@C_82_(CH_2_Ph) isomers are not known yet, and some of them may appear to be other regioisomers of Dy@*C*_3*v*_(8)-C_82_(CH_2_Ph).

In short, these estimations demonstrate that Dy@*C*_3*v*_(8)-C_82_ is the second most abundant Dy-EMF after Dy@*C*_2*v*_(9)-C_82_. Dy here is but a member of the lanthanide row. DFT calculations show that the relative stability of M^III^@*C*_3*v*_(8)-C_82_ does not change with the radius of M^3+^ from La^3+^ to Lu^3+^ (Figure 1b), and thus similar abundance can be expected. Indeed, compositions of benzylated EMF mixtures similar to Dy-EMFs were obtained for some other heavy lanthanides (Y, Gd, Tb, Ho, Er),^38,39^ suggesting that M^III^@*C*_3*v*_(8)-C_82_ is equally abundant for other rare-earth metals.

#### Position of the Metal in Dy@*C*_3*v*_(8)-C_82_(R)

**Figure 4:**
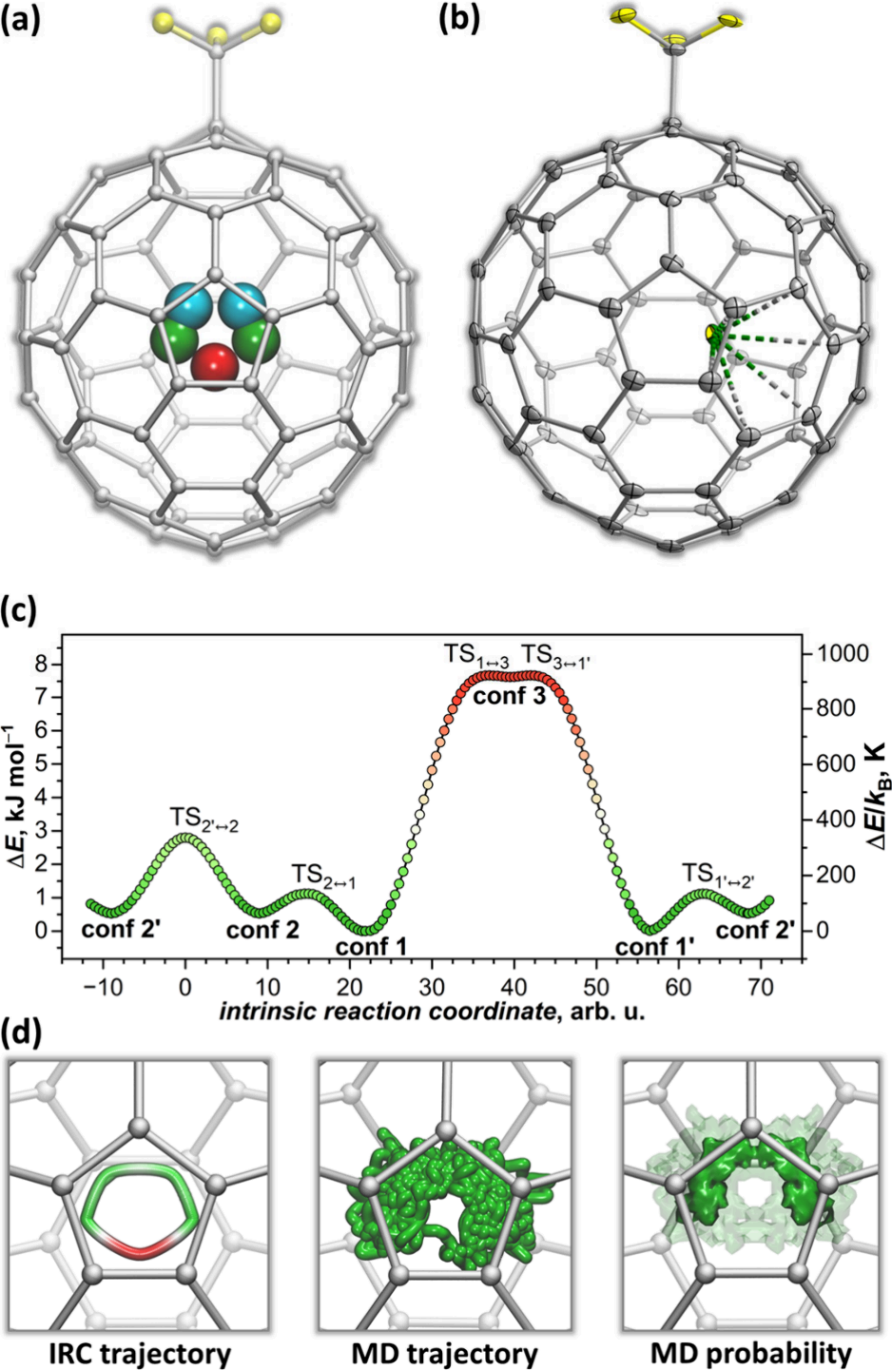
(a) Positions of Dy atom in three conformers of Dy@*C*_3*v*_(8)-C_82_(CF_3_) and their symmetry replica, giving five metal sites located around one pentagon; relative energies computed at the PBE0//PBE level are 0.0 kJ mol^–1^ for **conf 1** (green), 1.0 kJ mol^–1^ for **conf 2** (cyan), and 7.7 kJ mol^–1^ for **conf 3** (red). (b) SC-XRD structure of Dy@*C*_3*v*_(8)-C_82_(CF_3_) shown in the same orientation (F – yellow; C – gray; Dy – green). (c) Potential energy profile along the metal motion between conformers obtained in intrinsic reaction coordinate (IRC) calculations. (d) IRC trajectory for the metal motion corresponding to the energy profile in (c) (left), DFT-based molecular dynamics (MD) trajectory of metal atom (middle), and isosurfaces of the symmetrized probability density obtained from the MD trajectory (right, transparent isosurface – low probability, solid isosurface – high probability); DFT-based molecular dynamics trajectory of Y@*C*_3*v*_(8)-C_82_(CF_3_) was propagated for 50 ps at *T* = 300 K, calculations are performed at the PBE/TZ2P level with effective core potential for Y, oscillations of carbon atoms near equilibrium positions are not shown.

Fully ordered metal position in the SC-XRD structure of Dy@*C*_3*v*_(8)-C_82_(CF_3_) suggests the presence of only one energy minimum for a metal atom inside the fullerene cage. Since this is not very common for mono-EMFs, we analyzed possible metal positions in more details using density functional theory (DFT). Computations were first performed for Y@*C*_3*v*_(8)-C_82_(CF_3_) employing fast DFT code Priroda,^86,87^ PBE functional,^88^ and implemented TZ2P-quality basis set with effective core potential for Y. Optimization of 26 starting geometries with η^5^ or η^6^ coordination of metal atom to all inequivalent pentagons or hexagons of *C*_3*v*_(8)-C_82_(CF_3_) resulted in only three unique structures (conformers) shown in Figure 4a (see also Figure S10–S11). Together with their symmetry replica, they form five metal sites located near the pentagon/hexagon edges of one particular pentagon. The lowest-energy **conf 1** coincides with the SC-XRD structure (Figure 4a, green and Figure 4b). In **conf 2**, which is only 0.52 kJ mol^–1^ less stable, the metal position is the same as found in the nonfunctionalized Y@*C*_3*v*_(8)-C_82_ (Figure 4a, cyan). Finally, **conf 3** is 7.63 kJ mol^–1^ higher in energy and has the metal near the symmetry plane (Figure 4a, red). Replacement of CF_3_ with a benzyl group made **conf 2** more stable than **conf 1** by 1.5 kJ mol^–1^ and increased the relative energy of **conf 3** to 9.8 kJ mol^–1^ (Table S3).

In view of the small energy difference between Y@*C*_3*v*_(8)-C_82_(CF_3_) conformers, we also calculated transition states (TS) between them. TS_1↔2_ on the pathway between **conf 1** and **conf 2** is at 1.12 kJ mol^–1^, TS_2↔2′_ between two symmetry related replica of **conf 2** is at 2.80 kJ mol^–1^, while TS_1↔3_ is at 7.68 kJ mol^–1^. Intrinsic reaction coordinate (IRC) calculations were then performed to obtain the energy profile along the metal motion between the conformers (Figure 4c,d). These calculations show that the potential energy surface is very shallow and that the metal atom can experience a low-barrier fluxional motion around the perimeter of the coordinated pentagon. Particularly **conf 3** appears to be just a tiny dimple with the depth of only 0.05 kJ mol^–1^.

The flat potential surface also affects the lateral vibrational modes in which metal atoms oscillate parallel to the fullerene surface. Each metal atom in mono-EMF has two such modes.^89^ In all Y@*C*_3*v*_(8)-C_82_(CF_3_) conformers, one of them occurs near 60 cm^–1^ (43–46 cm^–1^ if Dy mass is used instead of Y). The other one, in which metal vibrates along the coordinate connecting conformers, occurs at lower frequencies and is conformer-dependent, from 42 (32) cm^–1^ in **conf 1** and 34 (26) cm^–1^ in **conf 2** to 15 (12) cm^–1^ in **conf 3** (the values in parentheses are for Dy). Harmonic approximation used in these calculations is probably too crude for these low frequencies, but regardless of the approximation, these modes should have large amplitudes, even at very low temperatures.

To obtain a dynamic picture of the metal motion, DFT-based molecular dynamics (MD) simulations were performed for *T* = 300 K. MD trajectory propagated for 50 ps demonstrated a fast motion of the metal between **conf 1**, **conf 2**, **conf 2′** and **conf 1′** with occasional but rare passing through **conf 3** (Figure 4d). Isosurface of the probability density shows that the contribution of **conf 3** to the physical properties should be small (Figure 4d). On the NMR time scale, the metal motion gives effective *C*_*s*_-symmetry of the molecule, which is observed in ^1^H NMR spectra of Dy@*C*_3*v*_(8)-C_82_(CH_2_Ph) (Figure 3a).

The fact that the SC-XRD structure has only one metal site is probably the effect of intermolecular interactions and in particular the coordination with NiOEP. Earlier we have shown that electrostatic interactions of EMF with NiOEP can change relative energies of EMF conformers by several kJ mol^–1^ and thus increase the preference of certain metal positions.^90^ It is worth noting that the Dy@*C*_3*v*_(8)-C_82_(CF_3_) molecule is oriented by Dy-binding site toward nitrogen atoms of NiOEP (Figure S7), and such metal-nitrogen orientation is also observed in SC-XRD structures of other EMFs.^36,37,91−94^

To ensure that the computational results are not affected by a particular functional, DFT code, or the use of Y to model Dy, we also optimized molecular structures of the three conformers of Dy@*C*_3*v*_(8)-C_82_(CF_3_) using PBE functional and 4f-in-core ECP basis with Orca suite,^95^ and then performed single-point calculations with PBE0 and B3LYP functionals. A high fidelity of the results was observed, the variation of the relative energies being less than 1–2 kJ mol^–1^ (Tables S3, S4).

#### Regioselectivity of Addition to [Dy@*C*_3*v*_(8)-C_82_]^−^

**Figure 5:**
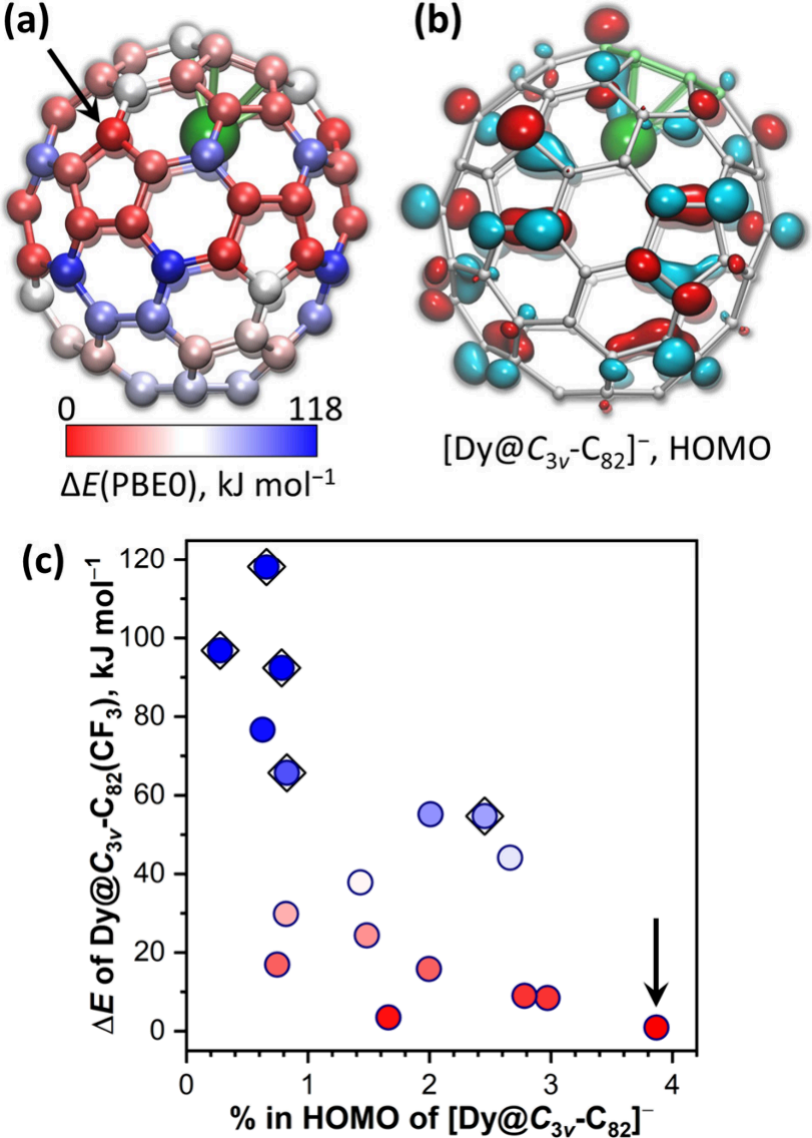
(a) Relative energies (Δ*E*) of 17 Dy@*C*_3*v*_(8)-C_82_(CF_3_) isomers computed at the PBE0//PBE level; each type of carbon atoms is color-coded in accordance with the relative energy of the Dy@C_82_(CF_3_) isomer, obtained by attaching CF_3_ group to it. (b) HOMO isosurface of [Dy@*C*_3*v*_(8)-C_82_]^−^. (c) Correlation between Δ*E* of Dy@*C*_3*v*_(8)-C_82_(CF_3_) isomers and maximum contribution of a given type of carbons to the HOMO in [Dy@*C*_3*v*_(8)-C_82_]^−^; [6,6,6] carbon atoms on triple-hexagon junctions are additionally marked by diamond symbols around the dots. Arrow in (a) and (c) mark the CF_3_ addition site in the experimentally determined structure of Dy@*C*_3*v*_(8)-C_82_(CF_3_).

In view of the 17 types of carbon atoms available in the *C*_3*v*_(8)-C_82_ cage, the high abundance of the HPLC fraction for one Dy@*C*_3*v*_(8)-C_82_(CH_2_Ph) regioisomer suggests that the addition of electrophilic groups to [Dy@*C*_3*v*_(8)-C_82_]^−^ may proceed with a high degree of regioselectivity. DFT calculations of 17 regioisomers of Dy@*C*_3*v*_(8)-C_82_(CF_3_) showed that the experimentally found structure has the lowest energy but several other isomers are nearly equally stable at the PBE0//PBE level. Relative energies of all isomers span 118 kJ mol^–1^ (Figure 5a, Figure S12, Table S4), but six most stable isomers are found within the range of only 17 kJ mol^–1^. In accordance with the rules developed for CF_3_ derivatives of fullerenes,^96^ attachment of the CF_3_ group to [6,6,6] carbon atoms on triple hexagon junctions results in the least stable isomers (Figure 5).

Since fullerene anions play the role of a nucleophile in both CH_2_Ph and CF_3_ addition, it is reasonable to suggest that the charge distribution in the anions should play a certain role in determining addition sites. Calculated QTAIM atomic charges in [Dy@*C*_3*v*_(8)-C_82_]^−^ did not show any correlation with the regioselectivity of CF_3_ or benzyl addition, but a loose correlation was found between the relative energy of Dy@C_82_(CF_3_) isomers and contributions of carbon atoms to the HOMO of [Dy@*C*_3*v*_(8)-C_82_]^−^ (Figure 5b,c). Importantly, the largest contribution to the HOMO was found for the CF_3_ and CH_2_Ph addition sites in the experimentally found adduct. Similar correlation between the CF_3_ addition site and the contribution to the HOMO of the fullerene anion was earlier found for Nd_2_@*D*_5*h*_-C_80_.^36^

### Magnetic Anisotropy of Dy Ion Induced by the Fullerene Cage

An increased interest to Dy-EMFs in the past decade is caused by the single-molecule magnetism (SMM) exhibited by many of them.^39,64,71,75,93,97−111^ SMM requires enhanced uniaxial ligand field, which in EMFs is realized when the endohedral cluster includes a nonmetal ion bonded to a lanthanide. Particularly high single-ion anisotropy was achieved in nitride^93,108,112,113^ and oxide^71,103−105^ clusterfullerenes, featuring N^3–^ and O^2–^ ions and short Dy–N and Dy–O bonds. Another successful approach to high-performance EMF-SMMs is based on lanthanide dimetallofullerenes with metal–metal bonds, which mediate a strong coupling between metal ions.^38,39,64,110,114−117^

Irrespective of the intracluster bonding, metal atoms in EMFs strongly interact with the carbon cage. On the one hand, there is a transfer of valence electrons to the fullerene, which is responsible for the substantial ionic contribution to the metal-cage bonding. On the other hand, there is also a significant metal d-orbital overlap with the carbon π-system.^53,118^ Simply put, the fullerene cage acts as a π-ligand akin to aromatic ligands in organolanthanide chemistry, such as cyclopentadiene or octatetraene. The difference is that the metal-fullerene bonding includes a more extended fragment of the fullerene cage, in which the interaction strength gradually decays with the M–C distance.^118^ The metal-cage interactions should naturally contribute to the ligand field (LF) and magnetic anisotropy of endohedral lanthanide ions, and their influence was observed as a variation of LF splitting in computationally studied conformers of Dy-clusterfullerenes.^71^ However, this contribution is considerably smaller than that of nonmetal ions or metal–metal bonds in EMF-SMMs, and it is hard to evaluate its role on the background of much stronger intracluster interactions.

Evidently, isolating the role of the metal-cage interaction in the magnetic anisotropy of endohedral metal ions requires the study of mono-EMFs. Magnetic properties of Dy@C_82_ were studied by SQUID magnetometry,^119^ Mössbauer spectroscopy,^120^ and X-ray magnetic circular dichroism (XMCD).^121−123^ Magnetic moments, determined in these works from magnetization curves or XMCD sum rule analysis, were considerably smaller than expected for a free Dy^3+^ ion. The deviations were ascribed to magnetic anisotropy induced by crystal-field states, but further details could not be obtained at that time. Besides, the results were affected by the interaction of Dy magnetic moment with unpaired spin on the fullerene cage. The story of molecular magnetism in mono-EMFs took a different turn with Dy@C_81_N, which has no unpaired electrons on the azafullerene cage.^109^ Despite the modest LF splitting and weak magnetic axiality predicted by CASSCF calculations and confirmed for the ground-state Kramers doublet (KD1) by EPR spectroscopy, Dy@C_81_N not only showed magnetic hysteresis but also exhibited opening until the highest temperature among all EMF-SMMs. In this work, attachment of a radical group to Dy@C_82_ similarly yields the closed-shell electronic structure of the fullerene and allows us to study the influence of the fullerene cage on magnetic anisotropy of the Dy^3+^ ion in mono-EMF.

#### *Ab Initio* Calculations

**Figure 6:**
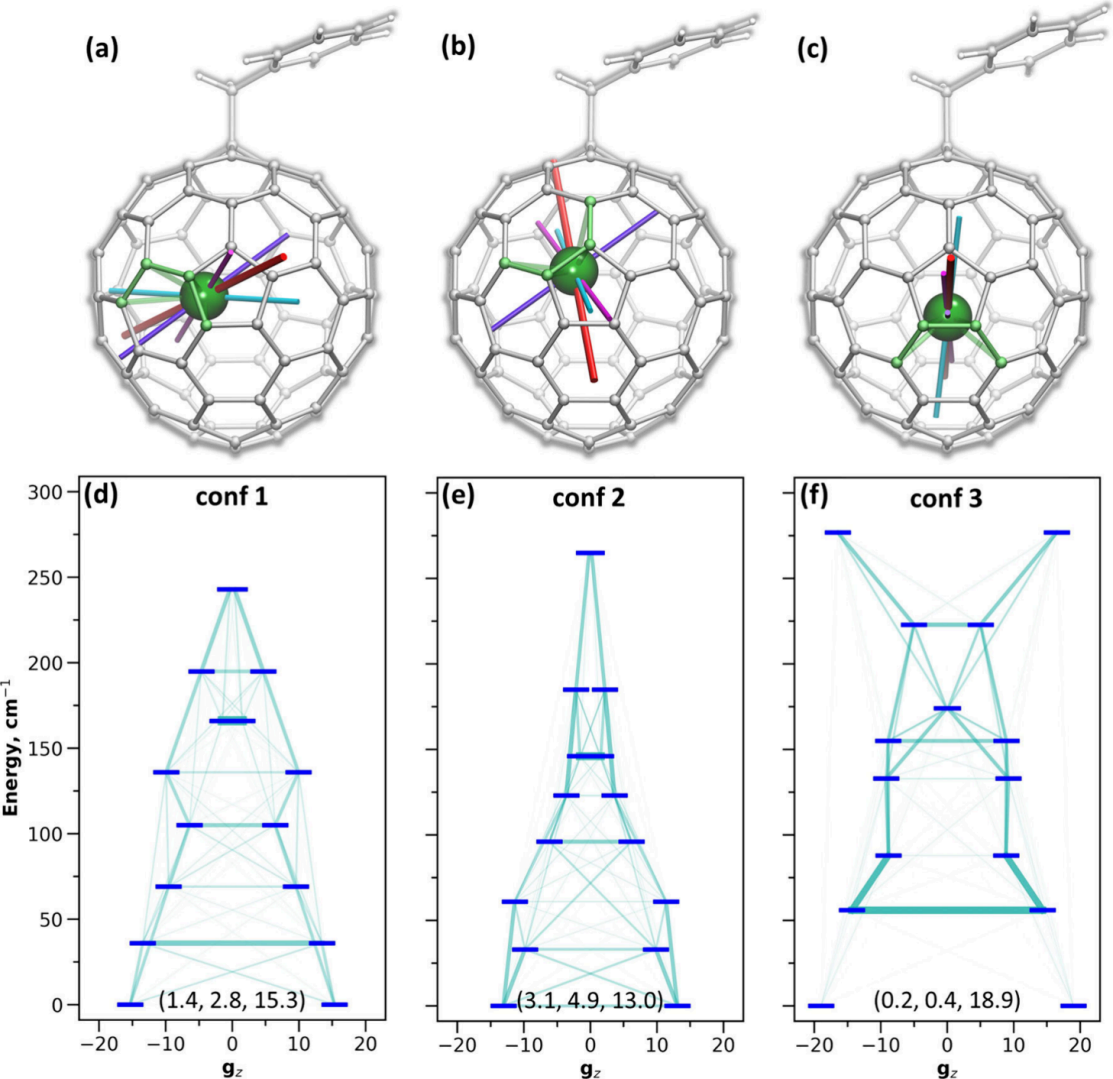
CASSCF calculations of ligand-field splitting in Dy@*C*_3*v*_(8)-C_82_(CH_2_Ph). (a–c) Orientations of principal *g*_*z*_ axes of four lowest Kramers doublets (KDs) in three conformers of Dy@*C*_3*v*_(8)-C_82_(CH_2_Ph): **conf 1** (a), **conf 2** (b), **conf 3** (c); principal *g*_*z*_-axes are shown in red (KD1), cyan (KD2), magenta (KD3), and violet (KD4), axis of the ground-state doublet KD1 is highlighted by a larger size, carbon atoms with Dy–C distance shorter than 2.5 Å are shown in light green. (d–f) Ligand-field splitting in Dy@C_82_(CH_2_Ph) conformers and projection of the *g*_*z*_ value of each KD on *g*_*z*_ direction of KD1; light blue lines visualize transition probabilities, numbers in parentheses are principal components of the pseudospin *g*-tensor, corresponding to the ground-state KD1.

Figure 6 visualizes the results of CASSCF/RASSI calculations^124,125^ for three conformers of Dy@*C*_3*v*_(8)-C_82_(CH_2_Ph). All of them show a moderate LF splitting of the ^6^*H*_15/2_ multiplet, from 246 cm^–1^ in **conf 1** to 285 cm^–1^ in **conf 3**. Position of the metal has a strong influence on the results, leading to a considerable difference between the conformers, although the local environment of Dy in these structures is very similar. On the other hand, replacement of CH_2_Ph group with CF_3_ did not noticeably affect the energies of doublet states in **conf 1** and **conf 2**, the variations of energy levels being less than 3 cm^–1^ (Table S5–S6, Figure S13). For **conf 3**, the changes appeared more pronounced, reaching up to 10 cm^–1^, but this effect is likely caused by the very shallow energy minimum, which leads to stronger changes of the coordination geometry (Table S7a,b). Further discussion will be dealt only with Dy@*C*_3*v*_(8)-C_82_(CH_2_Ph).

Composition of the doublet states has no distinct representation on the *m*_*J*_ basis (Tables S5–S7). The leading term in the ground-state Kramers doublet (KD1) of **conf 1** is 44% |±15/2⟩ followed by 22% |±11/2⟩ and 17% |±9/2⟩. In **conf 2**, the main terms in KD1 are 62% |±13/2⟩ and 9% |±11/2⟩. Higher-energy states are generally even more mixed. KD1 of **conf 3** shows a different character with 91% |±15/2⟩, but other KDs are strongly mixed, as well.

Pseudospin *g*-tensors of KDs also reflect the lack of well-defined quantization axes (Figure 6, Table S5–S7). The largest principal values (*g*_*z*_) for KD1 are 15.3 in **conf 1** and 13.0 in **conf 2**, while the transverse *g*_*x*,*y*_ values are substantially nonzero. Only **conf** 3 has a *g*-tensor with a *g*_*z*_ of 18.8 and small *g*_*x*,*y*_ values. On average, the *g*_*z*_ axis of KD1 tends to be parallel to the fullerene surface in the Dy-cage bonding site, but there is no clearer connection with the Dy-fullerene coordination geometry. Principal *z* axes of higher energy KDs have noticeably different orientations than that of KD1 (Figure 6a–c). In **conf 3**, orientations are restricted by the symmetry plane, but the angles between the *g*_*z*_ axes of different KDs are still substantial.

Net contributions of *Ô*_2_^*q*^, *Ô*_4_^*q*^, and *Ô*_6_^*q*^ operators in the LF splitting of Dy@*C*_3*v*_(8)-C_82_(CH_2_Ph) conformers are 32–34%, 11–13%, and 52–53%, respectively. More specifically, the net weight of axial LF parameters *B*_2_^0^, *B*_4_^0^, and *B*_6_^0^ is only 10% in **conf 1** and 16% in **conf 2**, but it increases to 41% in **conf 3** (note that unlike the net contribution of *B*_*k*_^*q*^ terms for all *q ≤ k*, the weight of individual terms depends on the choice of the quantization axis, which in this analysis was chosen as the *g*_*z*_ axis of KD1).

To summarize, the ligand field produced by the fullerene as a π-ligand is anything but axial. In the absence of structural elements, which would play a determining role on the presence and orientation of quantization axis, even small structural fluctuations lead to considerable changes of the orientation of principal axes and principal values of *g*-tensors. In combination with a flat potential energy surface, a low-barrier motion between **conf 1** and **conf 2**, and large amplitudes of low-frequency metal-based lateral modes, the magnetic anisotropy of Dy ion in Dy@*C*_3*v*_(8)-C_82_(CH_2_Ph) should be moderate and very fluid. Note that the size of the LF splitting and mixed compositions of the doublet states resembles those calculated for Dy@C_81_N,^109^ suggesting that this behavior is universal for mono-EMFs.

**Figure 7:**
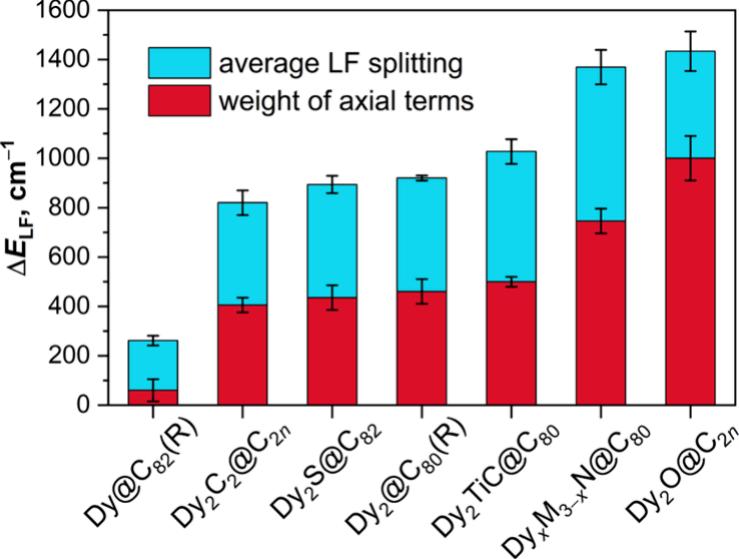
Average ligand-field splitting (Δ*E*_LF_) for Dy ions in different types of Dy-EMFs according to CASSCF calculations (full height of the bars) and the net weight of axial LF parameters *B*_2_^0^, *B*_4_^0^, and *B*_6_^0^ in the ligand field (red field in each bar). “Error” bars show the range of values in the studied molecules rather than standard deviations.

It is instructive to compare the magnetic anisotropy of Dy in mono-EMFs with clusterfullerenes and di-EMFs, which were extensively studied as SMMs. Unlike mono-EMFs, all of these molecules have a “strong” Dy–X bond, which imposes axial magnetic anisotropy with the quantization along the bond: Dy–N^3–^ in DyM_2_N@C_2*n*_ and Dy_2_MN@C_2*n*_, Dy–O^2–^ in Dy_2_O@C_2*n*_, Dy–S^2–^ in Dy_2_S@C_2*n*_, Dy–C^4–^ in Dy_2_TiC@C_80_, Dy–C_2_^2–^ in Dy_2_C_2_@C_2*n*_, or Dy–Dy in di-EMFs. Axiality is reflected in weights of axial terms in LF, which range from 50% to 70% (Figure 7). A ground state doublet is usually of pure Ising type with 99–100% weight of |±15/2⟩ and *g*-tensor with *g*_*z*_ of 19.8–19.9 and very small *g*_*x*__,*y*_ values (typically less than 10^–3^). Several higher-energy KDs usually also have relatively pure *m*_*J*_ composition with weights of leading terms of more than 90% and quantization axes nearly collinear with the ground-state KD. The size of LF splitting varies with X, from 1300–1500 cm^–1^ for O^2–^, 1300–1400 cm^–1^ for N^3–^, 1000–1200 cm^–1^ for C^4–^, 900–1000 cm^–1^ for S^2–^, to 800–900 cm^–1^ for C_2_^2–^ and 900–950 cm^–1^ for Dy–Dy bonds. Thus, even the weakest splitting is still considerably larger than 250–300 cm^–1^ induced solely by the fullerene cage (Figure 7).

Clusterfullerenes also often have conformers with different orientations of the endohedral unit and a flat energy surface, including the possibility of fluxional motion. But since Dy–X bonds are weakly affected by local details of the Dy-cage coordination geometry, single-ion magnetic anisotropy remains largely decoupled from the rotational dynamics of the endohedral cluster. The influence of Dy-cage interactions on the magnetic anisotropy is only seen in the variation of the LF splitting in the conformers and small misalignment of quantization axes from the Dy–X bond.

#### EPR Spectroscopy

**Figure 8:**
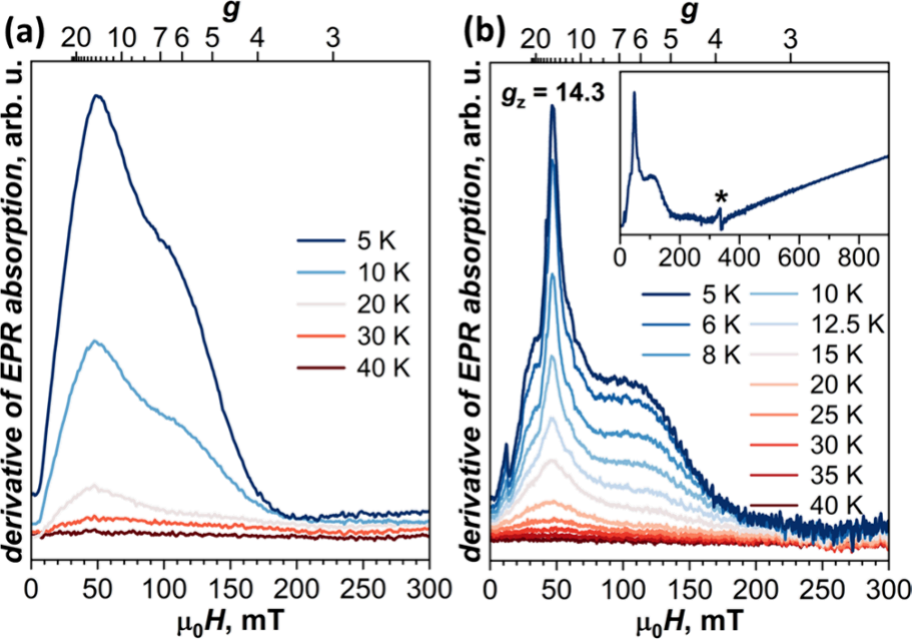
X-band EPR spectra of Dy@*C*_3*v*_(8)-C_82_(CH_2_Ph) measured at different temperatures: (a) powder sample, (b) glassy frozen solution in *o*-terphenyl. The inset in (b) shows the spectrum measured at 5 K in a broader magnetic field range, and the asterisk marks the signal of the resonator near *g* = 2. The spectra are baseline-corrected using the spectra measured at 50 K for (a) and 45 K for (b) and are shown with a small vertical offset. The spectrum in the inset in (b) is not baseline-corrected.

In the presence of highly axial ligand field, Dy^3+^ ion has well separated ground-state doublet described by pure *m*_*J*_ = ±15/2 functions and is not EPR active. However, when the ligand field is weak and nonaxial, KDs have mixed composition in *m*_*J*_ presentation, and transition matrix elements between components of KD1 can become sufficiently large to allow detection of EPR transitions. Figure 8a shows the X-band EPR spectra of powder Dy@*C*_3*v*_(8)-C_82_(CH_2_Ph). Between 5 and 20 K, the compound showed a broad asymmetric peak with a maximum at *g* = 13.4. The EPR signal decreased quickly with temperature and completely disappeared by 40 K. Due to the large width of the signal and the low field, in which it is observed, determination of the *g*-factor may be ambiguous. To reduce the line width, the fullerene was dissolved in *o*-terphenyl at 350 K and then flash-frozen in liquid nitrogen to obtain the glassy state. The spectrum became more resolved and showed a narrow peak at *g* = 14.3 (Figure 8b, Figure S14), which we interpret as *g*_*z*_. This value is close to *g*_*z*_ of 14.1 determined for Dy@C_81_N.^109^ The experimental *g*_*z*_ of Dy@*C*_3*v*_(8)-C_82_(CH_2_Ph) falls between theoretical *g*_*z*_ values for **conf 1** (15.3) and **conf 2** (13.0) and presumably corresponds to a mixture of both. The peak has several lower-intensity shoulders, which are likely caused by hyperfine interactions in ^161^Dy and ^163^Dy (*I* = 5/2 for both isotopes), but may also be caused by different positions of the metal atom. The line width is insufficiently narrow for a reliable analysis of these possibilities. The spectrum also has a broad peak between 50 and 200 mT, which may, in part, correspond to *g*_*x*_ and *g*_*y*_ components. We did not detect any other features in the spectrum, which could be assigned to *g*_*x*,*y*_, up to 900 mT (corresponding to *g* = 0.8).

#### SQUID Magnetometry

**Figure 9:**
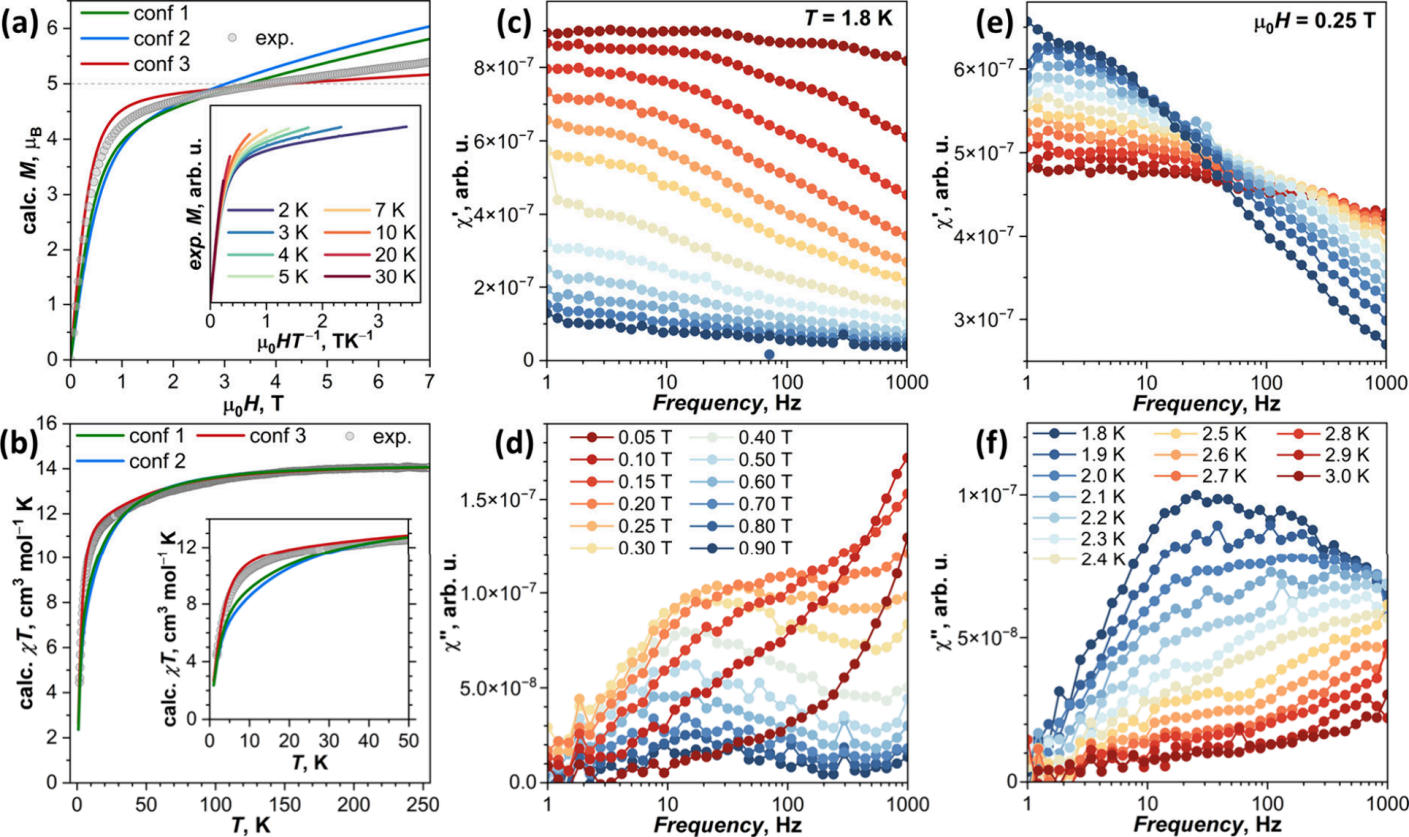
(a) Magnetization curve of powder Dy@C_82_(CH_2_Ph) measured at 1.8 K (dots) and overlaid with calculated curves for three conformers (solid lines); the inset shows magnetization as a function of quotient μ_0_*H*/*T* at different temperatures. (b) *χT* product of powder Dy@C_82_(CH_2_Ph) (dots) measured in the field of 1 T (χ is defined as *M*/*H*) and compared to calculations for three conformers (solid lines); the inset shows enlargement of the low-temperature range. (c, d) AC in-phase χ′ and out-of-phase χ″ magnetic susceptibility measured in different magnetic fields at the fixed temperature *T* = 1.8 K. (e, f) AC in-phase χ′ and out-of-phase χ″ magnetic susceptibility measured at different temperatures in the field of 0.25 T.

While EPR gives high-resolution information on the ground state doublet, magnetization behavior is also affected by other KDs, especially when the LF splitting is small. Figure 9a compares the isothermal magnetization curve of Dy@*C*_3*v*_(8)-C_82_(CH_2_Ph) measured at 1.8 K to curves of three conformers simulated using *ab initio* LF parameters. Magnetization of hypothetical isotropic Dy^3+^ would saturate at *g*_*J*_*J* = 10 μ_B_. For powder samples of axially anisotropic Dy^3+^ with considerable LF splitting, magnetization measured at 1.8 K saturates fast at 5 μ_B_. The factor of 0.5 with respect to 10 μ_B_ is caused by the random orientation of anisotropic magnetic moments in the powder. Calculated curves of **conf 1** and **conf 2** do not saturate until 7 T and show an intermediate magnetic moment of 6.0–6.2 μ_B_ at this field. The reason is the gradually increasing admixture of higher KDs to the wave function of KD1 with the increase of the magnetic field (note that thermal populations of higher KDs at 1.8 K should be negligible). This effect is known as van Vleck paramagnetism and usually leads to nearly linear growth of magnetization as a function of the magnetic field. The strongest effect of this factor is found for **conf 2**, which has the smallest LF splitting between low-energy KDs, an it is least pronounced in **conf 3**, which has twice larger gap between KD1 and KD2 and more pronounced axiality. The shape of the experimental curve passes between these two extremes. The van Vleck contribution seems to be overestimated by calculations, suggesting that the LF splitting is underestimated by CASSCF. Magnetization curves measured at higher temperatures do not coincide when presented as a function of the *HT*^–1^ quotient (Figure 9a, inset), indicating the influence of anisotropy and the contribution of several magnetic states. Calculated *χT* of **conf 1** and **conf 2** is considerably smaller than *χT* of **conf 3** at low temperature, but all curves coincide above 50 K, when more KDs become thermally populated. Experimental *χT* curve is closer to that of **conf 3**, similarly suggesting the underestimation of the LF splitting by theory (Figure 9b).

Dy@*C*_3*v*_(8)-C_82_(CH_2_Ph) did not show magnetic hysteresis in the DC measurements. To determine if the relaxation of magnetization can be observed on a faster time scale, AC measurements of in-phase and out-of-phase magnetic susceptibility (*χ′* and *χ″*) were performed (Figure 9c–f). At zero field and 1.8 K, no discernible *χ″* signal was observed, but the increase of the constant magnetic field resulted in the gradual development of the *χ″* peak, well distinguishable at 0.2–0.3 T (Figure 9d). Variable-temperature measurements performed in the field of 0.25 T demonstrated a fast decrease of the *χ″* signal above 1.8 K until its complete disappearance by 3 K (Figure 9f). The in-field magnetization relaxation time of Dy@*C*_3*v*_(8)-C_82_(CH_2_Ph) estimated from these measurements is near 0.02 s at 1.8 K. More detailed analysis of the relaxation mechanism can hardly be done based on the AC data because the *χ″* signal quickly becomes very broad above 1.8 K. It is sufficed to conclude that Dy@*C*_3*v*_(8)-C_82_(CH_2_Ph) behaves as a weak field-induced SMM. This magnetodynamic behavior is very different from that of Dy@C_81_N, which showed magnetic hysteresis up to at least 40 K,^109^ despite the moderate and nonaxial magnetic anisotropy akin to Dy@*C*_3*v*_(8)-C_82_(CH_2_Ph). The authors hypothesized that unexpectedly good SMM performance of Dy@C_81_N is rooted in the low phonon density of states, which isolates the magnetic moment of Dy from the lattice. Dy@*C*_3*v*_(8)-C_82_(R) adducts have similar metal-based modes but feature additional vibrations of the exohedral group. Whether this can be the reason for such a different SMM performance or it is the effect of coordination geometry requires further studies.

#### Paramagnetic NMR

Single-ion magnetic anisotropy also manifests itself in abnormal chemical shifts, as exhibited by paramagnetic compounds in NMR spectra. Experimental chemical shift can be presented as a sum of diamagnetic and paramagnetic contributions, δ^exp^*= δ*^dia^ + δ^para^. Paramagnetic shift itself is a combination of contact (Fermi) and pseudocontact (dipolar) terms, but given the relatively long distance between the Dy ion and CH_2_Ph or CF_3_ groups, the latter should be dominant in Dy@*C*_3*v*_(8)-C_82_(R) derivatives. Pseudocontact shift is a direct function of magnetic anisotropy (expressed via components of magnetic susceptibility tensor) and spatial position of nuclei with respect to the magnetic ion.^126^ For lanthanides, Bleaney showed that when LF splitting is smaller than the thermal energy, δ^para^ should have *T*^–2^ temperature dependence.^127^ Then, linearization of experimental chemical shifts in δ^exp^*-vs-T*^–2^ coordinates and extrapolation of the δ^exp^*= δ*^dia^ + *cT*^–2^ equation to *T*^–2^ = 0 gives estimation of diamagnetic shifts. This approximation was found to be adequate for ^1^H and ^13^C paramagnetic shifts in several Ce-EMFs.^17,128−132^ While the condition Δ*E*_LF_ < *k*_B_*T* is usually not fulfilled for lanthanide SMMs,^113,133−136^ the LF splitting in Dy mono-EMFs is close to *k*_B_*T*, and Bleaney’s approximation may become more applicable. As shown in Figure 3, the δ^exp^*-vs-T*^–2^ dependence of chemical shifts in Dy@*C*_3*v*_(8)-C_82_(R) is perfectly linear in the 258–318 K temperature range (*R*^2^ ≈ 0.9997). Diamagnetic shifts estimated from linear fits are 4.3 (b), 5.7 (c) and 6.2 (d) ppm for aromatic protons and 4.3 ppm for CH_2_ protons in the benzyl group, reasonably close to 7.2–7.8 ppm and 3.6–4.8 ppm in La@C_82_(CH_2_Ph) adducts.^137^ For the CF_3_ group in Dy@*C*_3*v*_(8)-C_82_(CF_3_), linear extrapolation gives δ^dia^ = −62.2 ppm, which is close to but still falls out of the expected range of −70 to −80 ppm. These results demonstrate that the anisotropy of the Dy ion in mono-EMFs is visible at room temperature in NMR chemical shifts, and its relatively small size ensures their *T*^–2^ temperature dependence.

## Conclusions

Monometallofullerene M^III^@C_82_ was one of the first discovered EMFs in the early 1990s and has been most abundantly produced and extensively studied ever since, but its isomeric composition had not been fully determined despite the numerous studies thereof. Here we demonstrated that the elusive isomer with a *C*_3*v*_(8)-C_82_ fullerene cage, whose high stability was predicted theoretically but never confirmed experimentally, is indeed formed in the arc-discharge synthesis. Furthermore, it appears to have one of the highest yields among EMFs, second only to the main M^III^@*C*_2*v*_(9)-C_82_ isomer. This surprising discovery is due to the low kinetic stability of M^III^@*C*_3*v*_(8)-C_82_, which prevented it from being extracted from the fullerene soot and structurally characterized in earlier studies. By using a combination of redox extraction with dimethylformamide and chemical functionalization of EMF anions, we obtained kinetically stable benzyl and trifluoromethyl adducts of Dy@*C*_3*v*_(8)-C_82_ and unambiguously determined its structure by single-crystal X-ray diffraction. The fact that such an abundant metallofullerene avoided being discovered for more than 30 years demonstrates that many other “unknown” EMFs may still be out there waiting to be found.

Having the monoadducts of Dy@C_82_ with well-defined molecular structure, we used the opportunity to address the influence of the fullerene cage and, in particular, metal-fullerene bonding on the magnetic anisotropy of Dy ion. The vast majority of Dy-EMFs, whose magnetic properties were studied during the past decade, were clusterfullerenes or dimetallofullerenes designed to have enhanced axial ligand field through a short and strongly polar Dy–X bond (where X is a nonmetal anion) or strong metal–metal interactions. The contribution of the fullerene cage as a π-ligand to the magnetic anisotropy of Dy has not been well studied because this effect is relatively small. Through a combination of *ab initio* calculations, EPR spectroscopy, and SQUID magnetometry, our work demonstrates that the fullerene cage alone produces moderate and generally nonaxial anisotropy in lanthanide ions. As a result, Dy@*C*_3*v*_(8)-C_82_(CH_2_Ph) is a weak field-induced SMM with relaxation of magnetization detectable only by AC magnetometry and only below 3 K. The lack of magnetic axiality implies that in most cases, the metal-cage bonding should be detrimental to the single-molecule magnetism in Dy-EMFs, although this negative effect is usually masked by much stronger Dy–X interactions. But as we approach the limits of magnetic anisotropy, which currently known EMFs with Dy–X bonds can offer, the enhancement of EMF-SMM performance requires considering other factors, and here the metal-cage bonding has hidden potential for a further progress. Our results show that the LF splitting and orientation of *g*-tensor principal axes strongly depend on the subtle details of metal-cage coordination geometry, which suggests that the geometry may be tuned to increase the axiality and thereby reinforce the axiality induced by intracluster interactions. The example of one conformer of Dy@*C*_3*v*_(8)-C_82_(CH_2_Ph) having a more pronounced magnetic axiality than that of the other two demonstrates this concept. Identifying metal-fullerene coordination moieties favoring the enhanced axiality and directing endohedral metal ions to required positions by an exohedral chemical modification or a supramolecular organization may become viable directions of future research.

## Supporting Information Available

Experimental and computational details, HPLC separation, additional computational results, additional crystallographic details, additional NMR and EPR spectra (PDF)
Cartesian coordinates of optimized structures (ZIP)
