# Supplementary material for: Thirty Years of Hide-and-Seek: Capturing Abundant but Elusive MIII@C3v(8)-C82 Isomer, and the Study of Magnetic Anisotropy Induced in Dy3+ Ion by the Fullerene π-Ligand
Source: J Am Chem Soc. 2024 Sep 2;146(36):25328–42. doi: 10.1021/jacs.4c10050 (PMC11403620; doi:10.1021/jacs.4c10050)
Supplement: Supplementary file 1 — ja4c10050_si_001.pdf [file ja4c10050_si_001.pdf]

30 years of hide-and-seek: capturing abundant but elusive M^{III}@C_{3v}(8)-C_{82} isomer, and the study of magnetic anisotropy induced in Dy^{3+} ion by the fullerene π-ligand

Wei Yang,†[a] Matheus Felipe de Souza Barbosa,†[a] Alexey Alfonsov,[a] Marco Rosenkranz,[a] Noel Israel,[a] Bernd Büchner,[a] Stanislav M. Avdoshenko,[a]* Fupin Liu,[b]* Alexey A. Popov[a]*

Supporting Information

Experimental and computational details S2

M^{III}@C_{82} isomers S4

Synthesis and separation of fullerene derivatives S5

Single-crystal X-ray diffraction S10

NMR spectra S13

DFT calculations of metal positions S14

Isomers of Dy@C_{3v}(8)-C_{82}(CF_3) S16

CASSCF calculations S18

Additional EPR data S20

Supplementary references S21

Experimental and Computational Details

Synthesis. Dy-EMFs were synthesized by direct current arc discharge method. Specifically, a mixture of Dy_2O_3 and graphite powder (C) with a molar ratio of Dy:C = 1:7.5 was filled into the core-drilled graphite rods, which were then evaporated in 180 mbar He atmosphere with a direct current of 100 A. Endohedral metallofullerenes were extracted from the synthesized soot by N, N-Dimethylformamide (DMF). The DMF solution of EMF anions were reacted with benzyl bromide or Umemoto reagent II, as described previously.[1, 2] The products were separated by high-performance liquid chromatography (HPLC) using toluene as the mobile phase.

HPLC. HPLC separation was performed for toluene solutions of fullerene and with toluene as an eluent, employing analytical or semipreparative COSMOSIL Buckyprep chromatographic columns (Nacalai Tesque) and Agilent 1260 Infinity II LC System. Recycling HPLC separation was performed using the Sunflow 100 system (SunChrome).

Mass-spectrometry. Matrix assisted laser desorption/ionization time-of-flight (MALDI-TOF) mass spectra were measured with a Bruker autoflex mass spectrometer.

UV-Vis-NIR. UV-vis-NIR absorption spectra were measured in carbon disulfide solution at room temperature with Shimadzu 3100 spectrophotometer.

Nuclear magnetic resonance (NMR) spectroscopy. ^{1}H and ^{19}F NMR spectra were measured with 500 MHz Avance II spectrometer (Bruker) in CS_2 solution.

X-ray diffraction. Single crystals were obtained by co-crystallization of Dy@C_{82}(CF_3) in toluene with nickel octaethylporphyrin (NiOEP) in benzene. X-ray diffraction data collection was carried out at the BL14.2 beamline (BESSY storage ring, Berlin-Adlershof, Germany).[3] XDSAPP2.0 suite was employed for data processing.[4, 5] The structure was solved by direct methods and refined by SHELXL-2018.[6] Hydrogen atoms were added geometrically and refined with a riding model. The crystal data are presented in Table S2.

Electron paramagnetic resonance (EPR) measurements were carried out on a pressed powder and o-terphenyl solution of Dy@C_{3v}(8)-C_{82}(CH_2Ph) at a fixed microwave frequency of ν = 9.56 GHz and at temperatures from 5 to 100 K using a commercial Bruker X-band spectrometer equipped with a ^{4}He gas flow cryostat from Oxford Instruments. The magnetic field was swept from 0 to 300 mT for most of the measurements. The EPR spectra were recorded as an absorption derivative.

Magnetization of powder samples was measured using a Quantum Design VSM MPMS3 magnetometer. In AC measurements, the oscillation amplitude was 2.5 Oe. Magnetic simulations were performed using PHI code[7] and employing powder-averaging to account for random orientation of molecules in experimentally-measured samples; single-ion ligand-field parameters were obtained in CASSCF calculations.

Computational studies. DFT calculations were first performed for Y analogs, molecular structures were optimized at the PBE[8] level using the fast DFT *Priroda* code[9, 10] with the implemented basis set of TZ2P quality with SBKJC-type effective core potential for Y atoms. The same code was used for transition state (TS) search and intrinsic reaction coordinate (IRC) calculations. DFT-level Born-Oppenheimer molecular dynamics were performed using in-house Python scripts and Atomic Simulation Environment libraries.[11] Energies and gradients at each time step were evaluated at the PBE/TZ2P level

with the *Priroda* code. The equation of motion was integrated for 25 ps with a step size of 1.0 fs, utilizing a canonical ensemble statistic and Nosé-Hoover thermostat for temperature control.

DFT calculations with Dy were performed using the *Orca* package.[12, 13] For lanthanides with non-zero orbital momentum, all-electron DFT calculations can give ambiguous results because of the non-single-determinant wavefunction. To avoid potential problems, we used 4f-in-core effective core potentials of ECP*XX*MWB by Dolg et al. with corresponding ECP*XX*MWB-II basis sets.[14, 15] For C and F atoms, def2-TZVPP basis was used.[16] The same code and basis sets were used for single-point calculations of energies with hybrid functionals PBE0 and B3LYP.

For consistency with earlier works, molecular structures for CASSCF calculations were optimized at the PBE/PAW level using the *VASP* code and recommended pseudopotentials with f-shell in-core treatment;[8, 17-20] the structures were very similar to those optimized with molecular codes. *Ab initio* energies and wave functions of Dy^{3+} LF multiplets have been calculated at the CASSCF/SO-RASSI level of theory using the quantum chemistry package OpenMOLCAS[21] and SINGLE_ANISO module.[22] The basis sets were ANO-RCC-VTZP for Dy and ANO-RCC-VDZP for other elements. To make axial term decomposition, we used ligand-field parameters calculated with the OPEN_SINGLE_ANISO module to construct a crystal-field Hamiltonian. Having constructed the Hamiltonian for different compositions, such as $\hat{O}_2^q$ only or $\hat{O}_2^q$+$\hat{O}_4^q$, etc., we estimated the weights of these compositions in terms of Euclidean norms.[22]

Molecular structures, trajectories, and isosurfaces were visualized with VMD.[23]

Molecular structures and relative energies of $M^{III}@C_{82}$ isomers

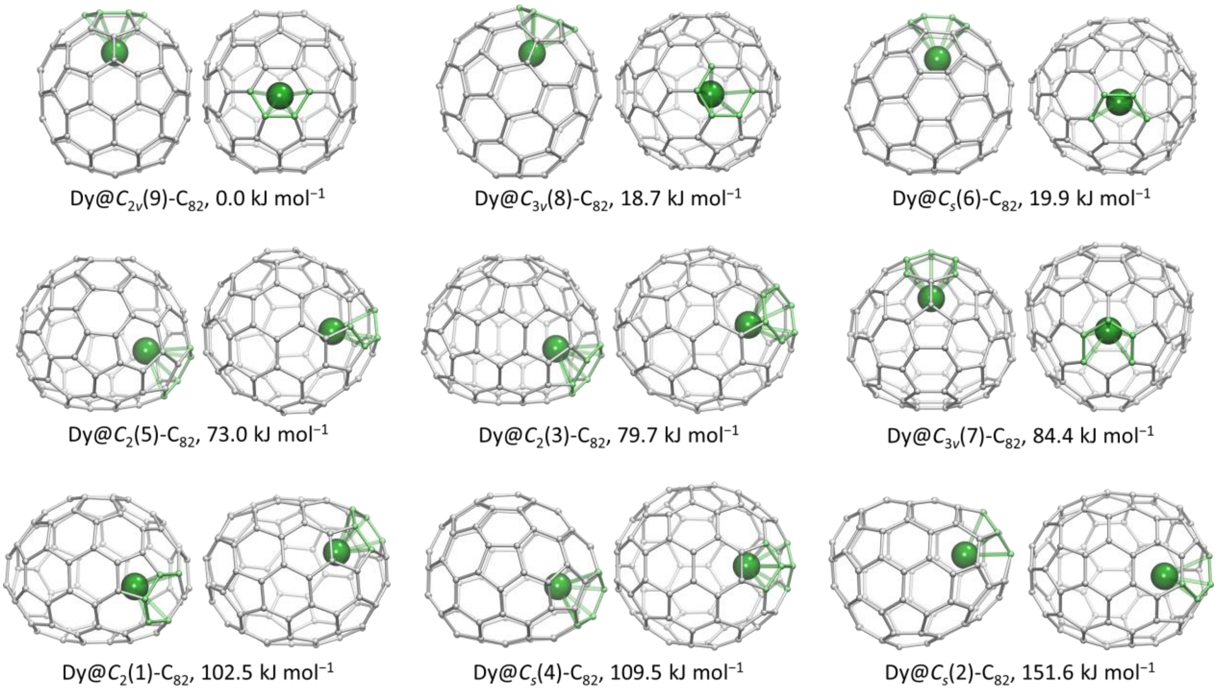

Figure S1. DFT-optimized molecular structures of Dy@C_{82} isomers and their relative energies (PBE0//PBE level). Each structure is shown in two orientations.

Table S1. Relative energies (kJ mol^{-1}) of $M^{III}@C_{82}$ isomers

**$M^{III}@C_{82}$ isomer**	**PBE[a]**	**PBE[a]**	**PBE[a]**	**PBE[a]**	**PBE[b]**	**PBE0//PBE[c]**
	M = Sc	M = Lu	M = La	M = Y	M = Dy	M = Dy
**C_{2v}(9)**	0.0	0.0	0.0	0.0	0.0	0.0
**C_{3v}(8)**	21.1	20.1	17.9	19.6	19.6	18.7
**C_s(6)**	18.5	19.2	20.9	19.4	19.9	19.9
**C_2(5)**	45.5	52.4	76.0	58.7	59.6	73.0
**C_2(3)**	51.0	59.3	87.8	67.9	67.1	79.7
**C_{3v}(7)**	73.6	77.9	83.2	78.1	79.6	84.4
**C_2(1)**	74.7	81.0	103.7	88.1	87.7	102.5
**C_s(4)**	71.8	80.2	113.3	89.0	89.2	109.5
**C_s(2)**	114.0	120.6	149.2	130.3	129.0	151.6

[a] PBE/TZ2P with SBKJC-type effective core potential for metals, Priroda code

[b] PBE/def2-TZVP with 4f-in-core effective core potential for Dy, Orca code

[c] point energy calculations using PBE0 functional and PBE-optimized geometry

HPLC separation

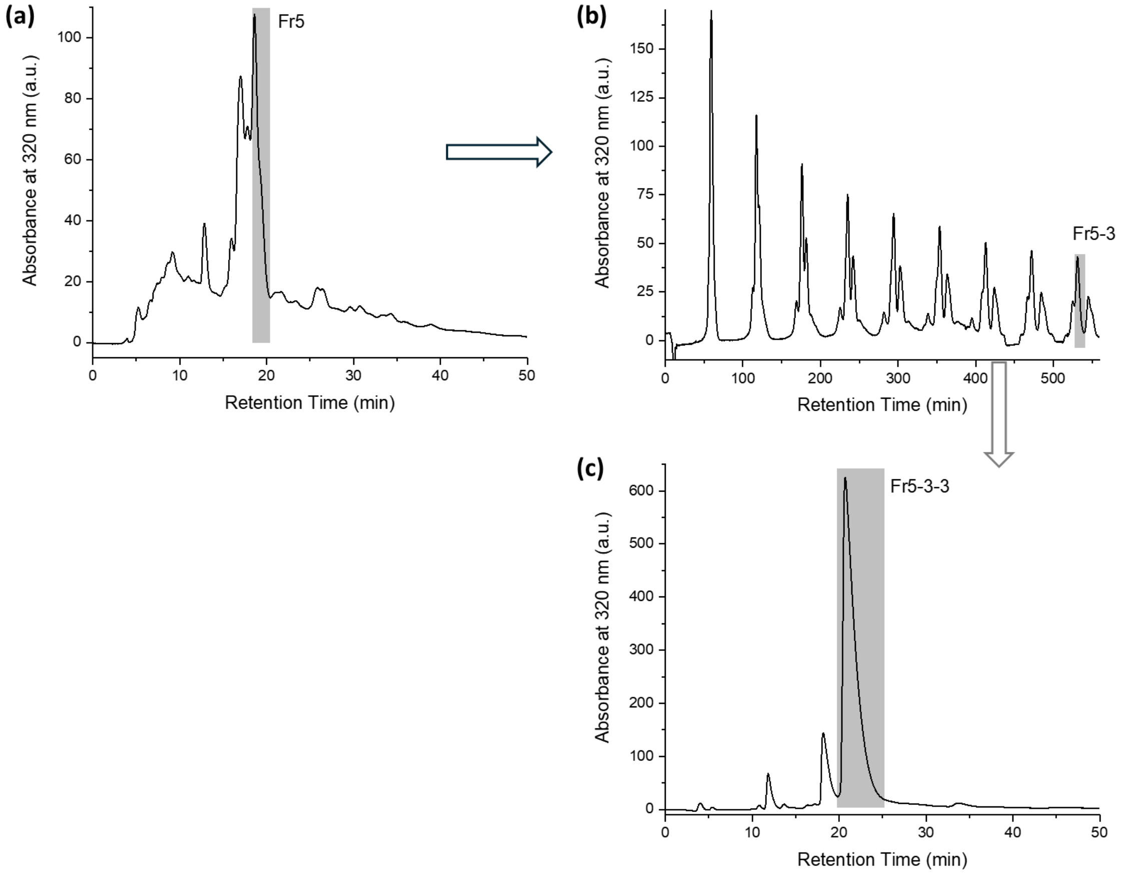

Figure S2a. The HPLC separation procedure of Dy@C_{82}(CH_2Ph). **(a)** HPLC profile of the mixture of benzyl-derivatized Dy-EMFs. The highlighted fraction marked Fr5 contained Dy@C_{82}(CH_2Ph) and Dy_2@C_{80}(CH_2Ph) derivatives. HPLC conditions: linear combination of two 4.6 × 250 mm Buckyprep columns; flow rate 1.6 mL/min; injection volume 800 μL; toluene as eluent; 40 °C. **(b)** Second step HPLC separation profile of Fr5 containing Dy@C_{82}(CH_2Ph) with recycling mode (10 × 250 mm Buckyprep column; flow rate 2 mL/min; injection volume 4.5 mL; toluene as eluent). **(c)** Third step HPLC separation of Fr5-3 containing Dy@C_{82}(CH_2Ph) to get the pure compound as Fr5-3-3 (4.6 × 250 mm Buckyprep-D column; flow rate 1 mL/min; injection volume 0.5 mL; toluene as eluent; 40 °C).

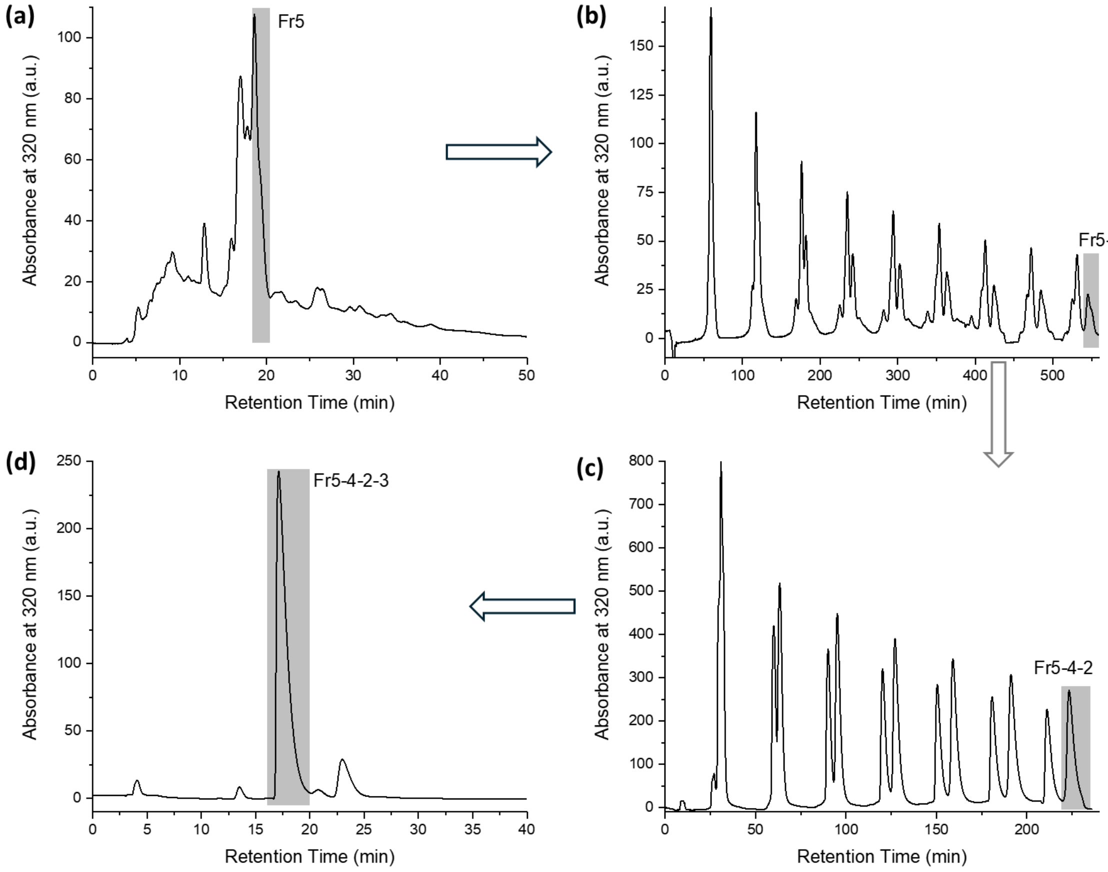

Figure S2b. The HPLC separation procedure of $Dy_2@C_{80}(CH_2Ph)$. **(a)** HPLC profile of the mixture of benzyl-derivatized Dy-EMFs. The highlighted fraction marked Fr5 contained $Dy@C_{82}(CH_2Ph)$ and $Dy_2@C_{80}(CH_2Ph)$ derivatives. HPLC conditions: linear combination of two 4.6 × 250 mm Buckyprep columns; flow rate 1.6 mL/min; injection volume 800 μL; toluene as eluent; 40 °C. **(b)** Second step HPLC separation profile of Fr5 containing $Dy@C_{82}(CH_2Ph)$ and $Dy_2@C_{80}(CH_2Ph)$ with recycling mode (10 × 250 mm Buckyprep column; flow rate 2 mL/min; injection volume 4.5 mL; toluene as eluent). **(c)** Recycling HPLC profile of fraction Fr5-4 containing $Dy_2@C_{80}(CH_2Ph)$ (10 × 250 mm Buckyprep-M column; flow rate 2 mL/min; injection volume 4.5 mL; toluene as eluent). **(d)** HPLC profile of fraction Fr5-4-2 (4.6 × 250 mm Buckyprep-D column; flow rate 1.0 mL/min; injection volume 0.5 mL; toluene as eluent; 40 °C). Pure $Dy_2@C_{80}(CH_2Ph)$ was obtained as fraction Fr5-4-2-3. The yield ratio of $Dy@C_{82}(CH_2Ph)/Dy_2@C_{80}(CH_2Ph)$ is estimated as 2.3 from the HPLC peak area.

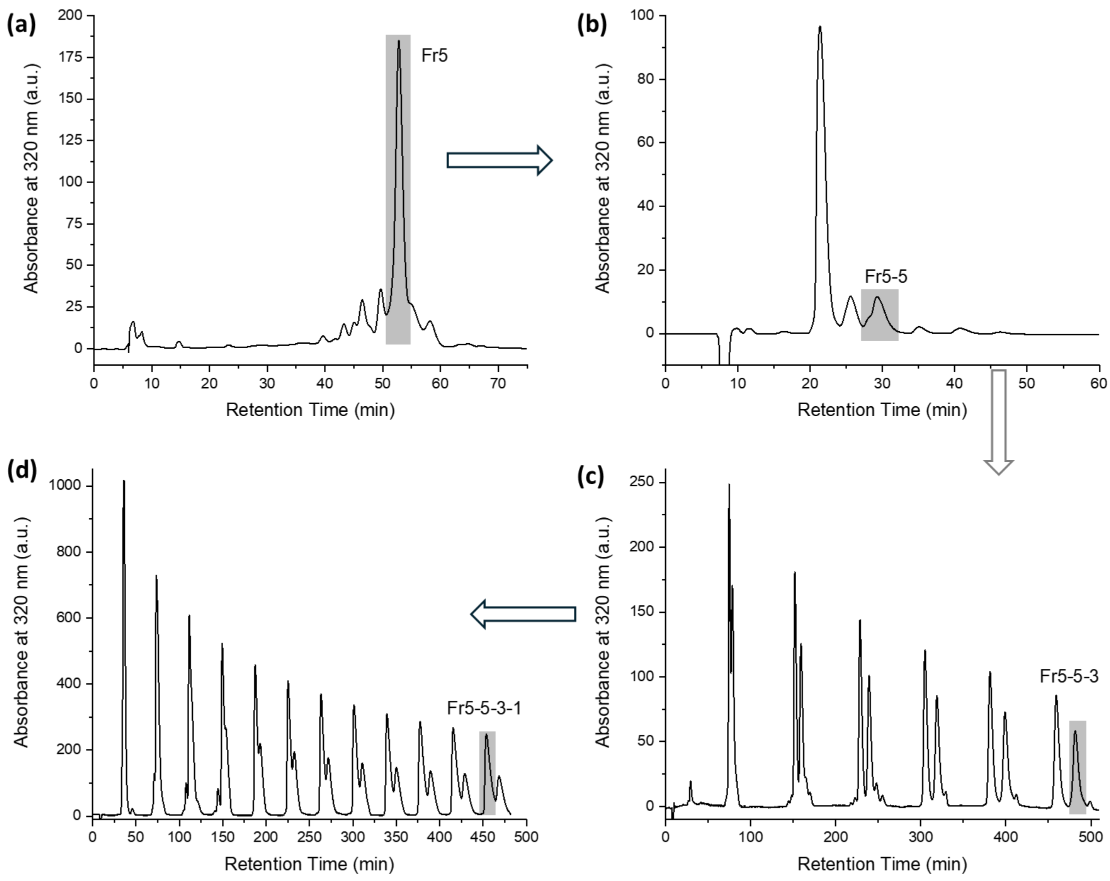

Figure S3a. The HPLC separation procedure of Dy@C_{82}(CF_3). **(a)** HPLC profile of the mixture of CF_3-derivatized Dy-EMFs. The highlighted fraction marked Fr5 contained Dy@C_{82}(CF_3)/Dy_2@C_{80}(CF_3) derivatives. HPLC conditions: linear combination of two 10 × 250 mm Buckyprep columns, flow rate 5 mL/min; injection volume 4.5 mL; toluene as eluent; 40 °C. **(b)** Second step HPLC separation profile of Fr5 containing Dy@C_{82}(CF_3) and Dy_2@C_{80}(CF_3) (linear combination of two 10 × 250 mm Buckyprep-D columns, flow rate 4 mL/min; injection volume 4.5 mL; toluene as eluent; 40 °C). **(c)** Third step HPLC separation of Fr5-5 containing Dy@C_{82}(CF_3) with recycling mode (10 × 250 mm Buckyprep column; flow rate 2 mL/min; injection volume 4.5 mL; toluene as eluent). **(d)** Recylcing HPLC profile of fraction Fr5-5-3 containing Dy@C_{82}(CF_3) (10 × 250 mm Buckyprep-M column; flow rate 2 mL/min; injection volume 4.5 mL; toluene as eluent). Pure Dy@C_{82}(CF_3) was obtained as fraction Fr5-5-3-1.

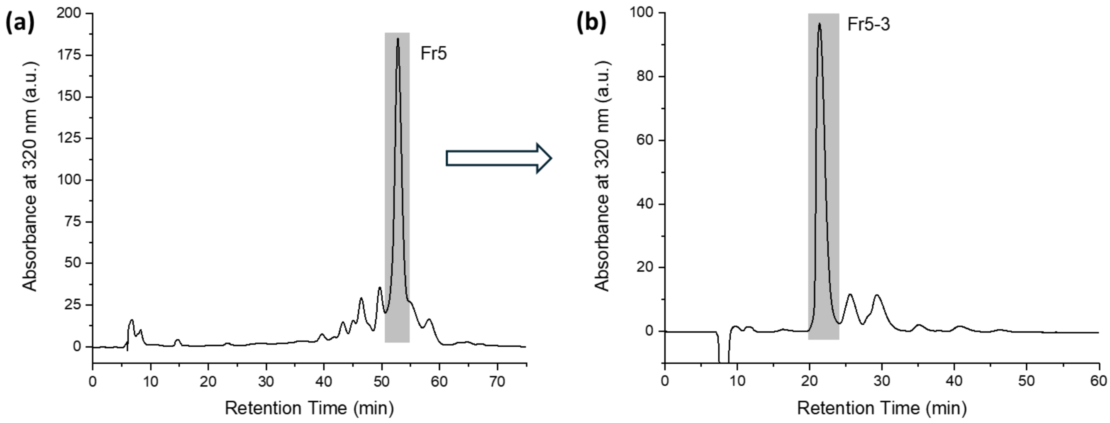

Figure S3b. The HPLC separation procedure of $Dy_2@C_{80}(CF_3)$. **(a)** HPLC profile of the mixture of CF_3-derivatized Dy-EMFs. The highlighted fraction marked Fr5 contained $Dy@C_{82}(CF_3)/Dy_2@C_{80}(CF_3)$ derivatives. HPLC conditions: linear combination of two 10 × 250 mm Buckyprep columns, flow rate 5 mL/min; injection volume 4.5 mL; toluene as eluent; 40 °C. **(b)** Second step HPLC separation profile of Fr5 containing $Dy@C_{82}(CF_3)$ and $Dy_2@C_{80}(CF_3)$ (linear combination of two 10 × 250 mm Buckyprep-D columns, flow rate 4 mL/min; injection volume 4.5 mL; toluene as eluent; 40 °C). Pure $Dy_2@C_{80}(CF_3)$ was obtained as fraction Fr5-3. The yield ratio of $Dy@C_{82}(CF_3)/Dy_2@C_{80}(CF_3)$ is estimated as 0.05 from the HPLC peak area.

Mass-spectral analysis

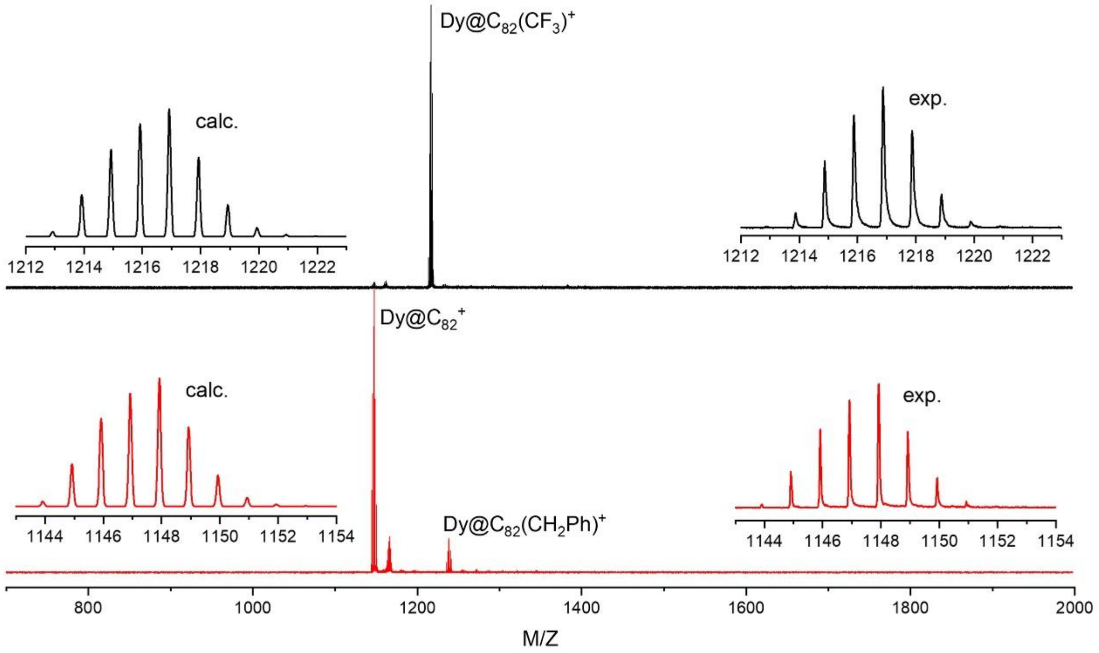

Figure S4. Matrix assisted laser desorption/ionization time-of-flight (MALDI TOF) mass spectra of Dy@C_{82}(CF_3) and Dy@C_{82}(CH_2Ph). Positive ion reflection mode was applied. The matrix is 1,1,4,4-Tetraphenyl-1,3-butadiene (TPBD). Fullerene derivatives often show fragmentation in MALD conditions. Dy@C_{82}(CF_3) is quite stable with respect to fragmentation, giving molecular ion as the main signal. But molecular ion of Dy@C_{82}(CH_2Ph) can be detected as well, but its fragmentation is much stronger, resulting in Dy@C_{82}^+ as the main signal.

Single-crystal X-ray diffraction

The asymmetric unit contains one NiOEP, one fullerene molecule, and two toluene molecules. Like the prevalent behaviour of fullerene-NiOEP co-crystals, the fullerene molecules were hold by NiOEP as shown in Figure S5. All molecule are fully ordered, the symmetry of fullerene cage is determined as C_{3v}(8)-C_{82}. The encapsulated Dy is fully ordered, locating beneath one hexagon as shown in Figure S6. The CF_3 functional group attaches to a hexagon-hexagon-pentagon (HHP) junction sitting on the mirror symmetry of fullerene cage, resulting in the C_s symmetry of C_{3v}(8)-$C_{82}(CF_3)$.

Table S2. Crystal data_Dy@C_{3v}(8)-$C_{82}(CF_3)$

Crystal	Dy@$C_{82}(CF_3)$·NiOEP·2C_7H_8
**Formula**	$C_{133}H_{60}DyF_3N_4Ni$
**Formula weight**	1992.06
**Color, habit**	Black, block
**Crystal system**	triclinic
**Space group**	$P\bar{1}$
***a*, Å**	14.720(3)
***b*, Å**	14.730(3)
***c*, Å**	20.600(4)
***α*, deg**	90.70(3)
***β*, deg**	95.10(3)
***γ*, deg**	118.44(3)
**Volume, Å^3**	3904.4(17)
Z	2
***T*, K**	100
**Radiation (λ, Å)**	Synchrotron Radiation (0.77977)
**Unique data (R_{int})**	23057 (0.0497)
**Parameters**	1290
**Restraints**	6
**Observed data ($I > 2\sigma(I)$)**	22289
**R_1^a (observed data)**	0.0766
**wR_2^b (all data)**	0.2453
**CCDC NO.**	2370148

[a]For data with $I > 2\sigma(I)$, $R_1 = \frac{\sum||F_O|-|F_C||}{\sum|F_O|}$. [b]For all data, $wR_2 = \sqrt{\frac{\sum[w(F_O^2-F_C^2)^2]}{\sum[w(F_O^2)^2]}}$.

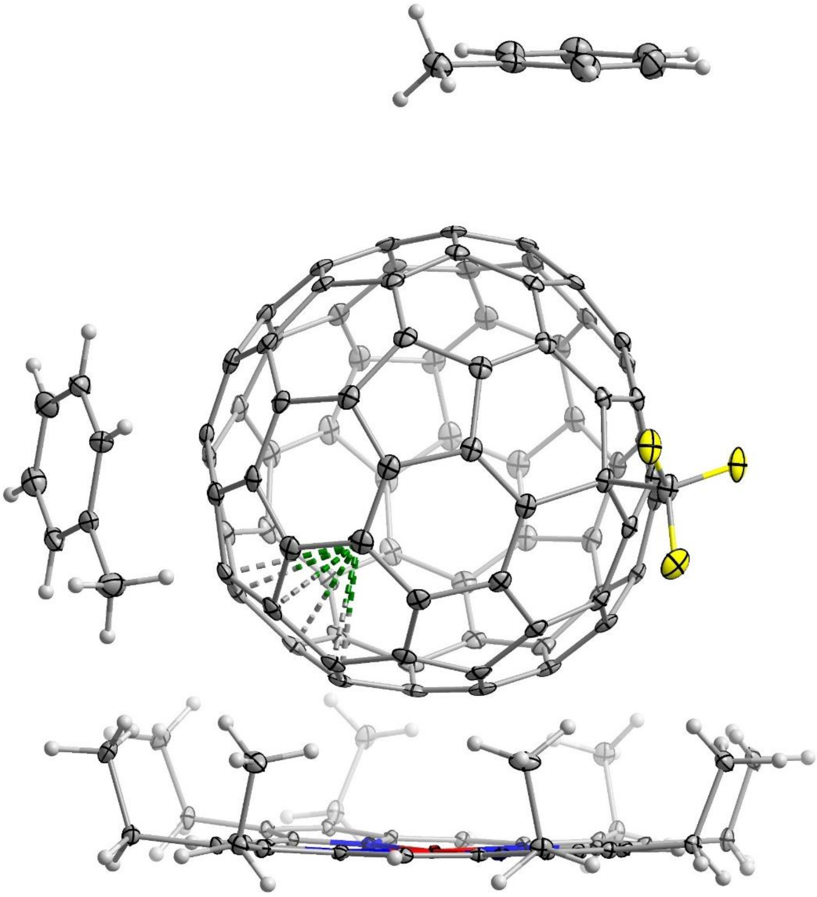

Figure S5. Structure of Dy@C_{3v}(8)-$C_{82}(CF_3)$·NiOEP·2Toluene. The thermal ellipsoid probability was set at 30%. Color code: grey for C, white for H, blue for N, green for Dy, and red for Ni.

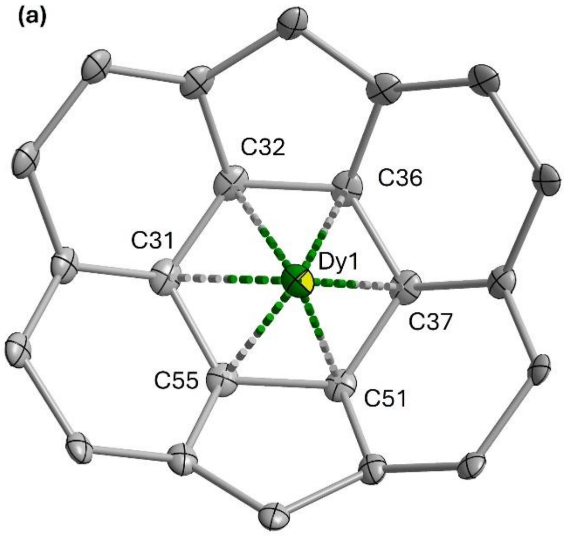

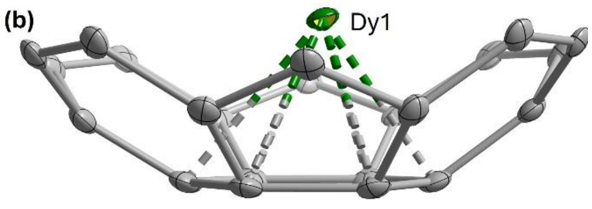

Dy–C bond	Bond length (Å)
Dy1–C51	2.319(6)
Dy1–C37	2.365(6)
Dy1–C55	2.401(6)
Dy1–C36	2.456(6)
Dy1–C31	2.551(7)
Dy1–C32	2.555(6)

Figure S6. Structure of Dy@C_{3v}(8)-$C_{82}(CF_3)$·NiOEP·2Toluene showing the Dy coordinated fragment viewed from to perpendicular directions. The Dy–C bond lengths were compiled in the table. The distance between Dy and the coordinated hexagon is 1.9527(6) Å. The thermal ellipsoid probability was set at 30%. Color code: grey for C and green for Dy.

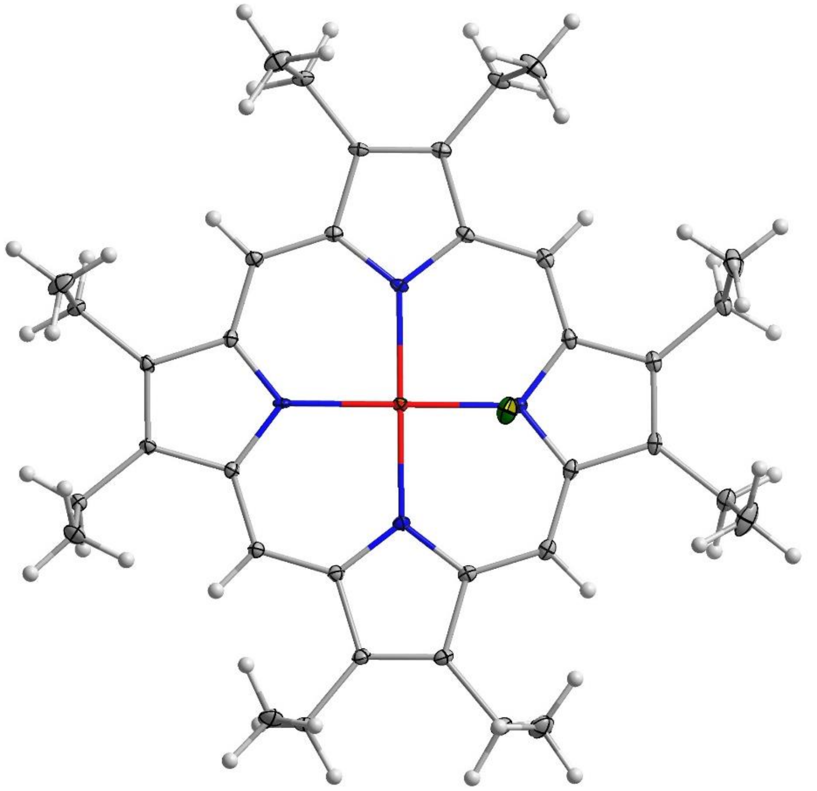

Figure S7. Structure of Dy@C_{3v}(8)-$C_{82}(CF_3)$·NiOEP·2Toluene. Solvent molecules and fullerene cage carbons are omitted for clarity to show the metal positions relative to the NiOEP. The thermal ellipsoid probability was set at 30%. Color code: grey for C, white for H, blue for N, green for Dy, and red for Ni.

NMR spectra

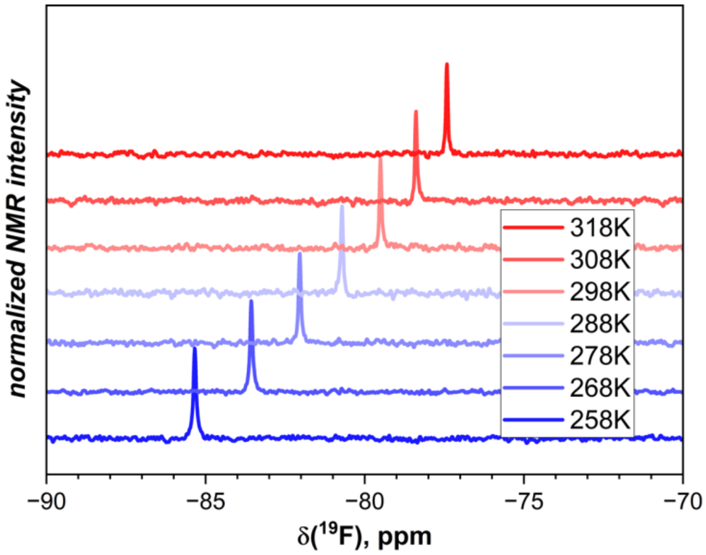

Figure S8. Variable-temperature ^{19}F NMR spectra of Dy@C_{3v}(8)-C_{82}(CF_3) in CS_2

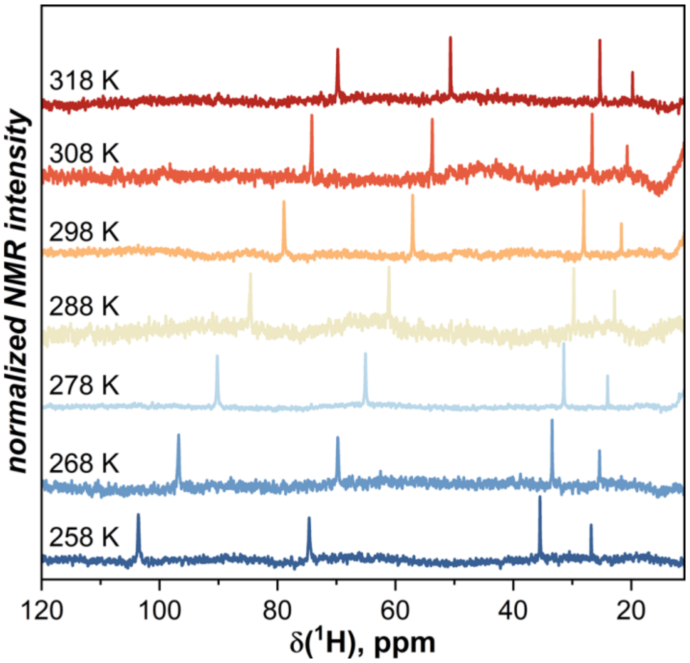

Figure S9. Variable-temperature ^{1}H NMR spectra of Dy@C_{3v}(8)-C_{82}(CH_2Ph) in CS_2

Calculations of metal position

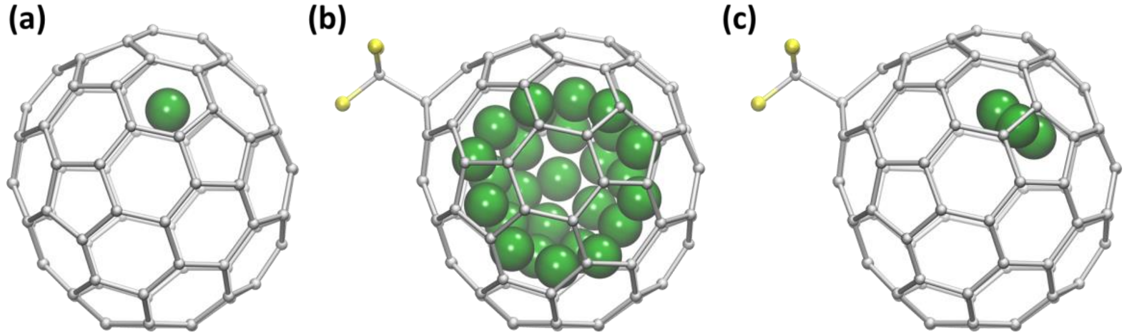

Figure S10. (a) DFT-optimized molecular structure of Dy@C_{3v}(8)-C_{82}. (b) C_{3v}(8)-C_{82}(CF_3) with CF_3 positions as in the SC-XRD structure and 26 starting positions of metal atoms used in computational survey of possible conformers. During optimization, metal atoms migrated to more preferable positions. (c) Three unique conformers obtained after optimization of all structures shown in (b).

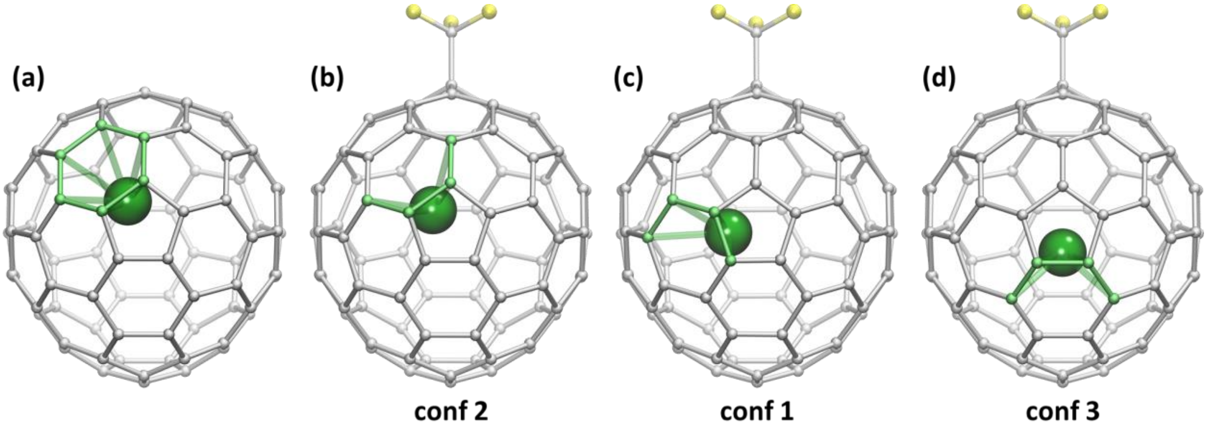

Figure S11. (a) DFT-optimized molecular structure of Dy@C_{3v}(8)-C_{82}. (b)-(c) DFT-optimized molecular structures of three conformers of Dy@C_{3v}(8)-C_{82}(CF_3). Carbon atoms with Dy–C distance less than 2.5 Å are shown in light green.

Table S3. Relative energies (kJ mol^{-1}) of conformers of M@C_{3v}(8)-C_{82}(CF_3) and transition states between them

	Y opt PBE[a]	Dy SP (Y), PBE[b]	Dy SP (Y), PBE0[b]	Dy SP (Y), B3LYP[b]	Dy opt PBE[c]	Dy PBE0//PBE[d]
**conf 1**	0.00	0.00	0.00	0.00	0.00	0.00
**conf 2**	0.53	1.25	0.94	1.11	1.24	0.99
**conf 3**	7.63	7.47	7.68	7.63	7.42	7.67
**$TS_{1\leftrightarrow 2}$**	1.12	1.25	0.92	0.89		
**$TS_{2\leftrightarrow 2'}$**	2.80	3.60	2.70	2.76		
**$TS_{1\leftrightarrow 3}$**	7.68	7.60	7.70	7.61		

[a] opt PBE – optimized at the PBE/TZ2P level with SBKJC-type ECP for Y, Priroda code;

[b] Single-point calculations using geometry optimized for Y, def2-TZVPP basis, basis set for Dy includes Dolg's 4f-in-core ECP, Orca code;

[c] optimized at the PBE/def-TZVPP level with Dolg's 4f-in-core ECP for Dy, Orca code;

[d] Single-point calculations using geometry optimized at the PBE/def2-TZVPP level, basis set for Dy includes Dolg's 4f-in-core ECP, Orca code;

Table S3. Relative energies (kJ mol^{-1}) of conformers of M@C_{3v}(8)-C_{82}(CH_2Ph)[a]

	Y<br>opt PBE[b]	Dy<br>opt PBE[c]	Dy<br>PBE0//PBE[d]	Dy<br>B3LYP//PBE[e]	Dy<br>B3LYP-D3BJ//PBE[f]
**conf 1-hex1**	1.91	0.73	1.67	1.29	1.61
**conf 1-hex2**	1.71	0.42	1.18	0.86	0.97
**conf 1-pent**	1.47	0.00	0.77	0.30	1.63
**conf 2-hex1**	0.77	0.69	0.81	0.73	0.83
**conf 2-hex2**	0.00	0.01	0.00	0.01	0.00
**conf 2-pent**	0.39	0.13	0.31	0.00	1.20
**conf 3-hex1**	10.38	8.65	9.72	9.34	8.90
**conf 3-hex2**	10.38	8.65	9.69	9.32	8.88
**conf 3-pent**	9.84	7.75	8.76	8.26	8.92

[a] for each metal position, rotation of benzyl group around the $C_{fullerene}$–C(H_2) bond gives three additional rotamers, one with Ph group above the fullerene pentagon, and two with Ph group above fullerene hexagons;

[b] opt PBE – optimized at the PBE/TZ2P level with SBKJC-type ECP for Y, Priroda code;

[c] optimized at the PBE/def-TZVPP level with 4f-in-core ECP for Dy, Orca code;

[d] Single-point calculations with PBE0 functional using geometry optimized at the PBE/def2-TZVPP level, basis set for Dy includes Dolg's 4f-in-core ECP, Orca code;

[e] Single-point calculations with B3LYP functional using geometry optimized at the PBE/def2-TZVPP level, basis set for Dy includes Dolg's 4f-in-core ECP, Orca code;

[f] Single-point calculations with B3LYP functional and D3BJ correction using geometry optimized at the PBE/def2-TZVPP level, basis set for Dy includes Dolg's 4f-in-core ECP, Orca code;

Isomers of Dy@C_{3v}(8)-$C_{82}(CF_3)$

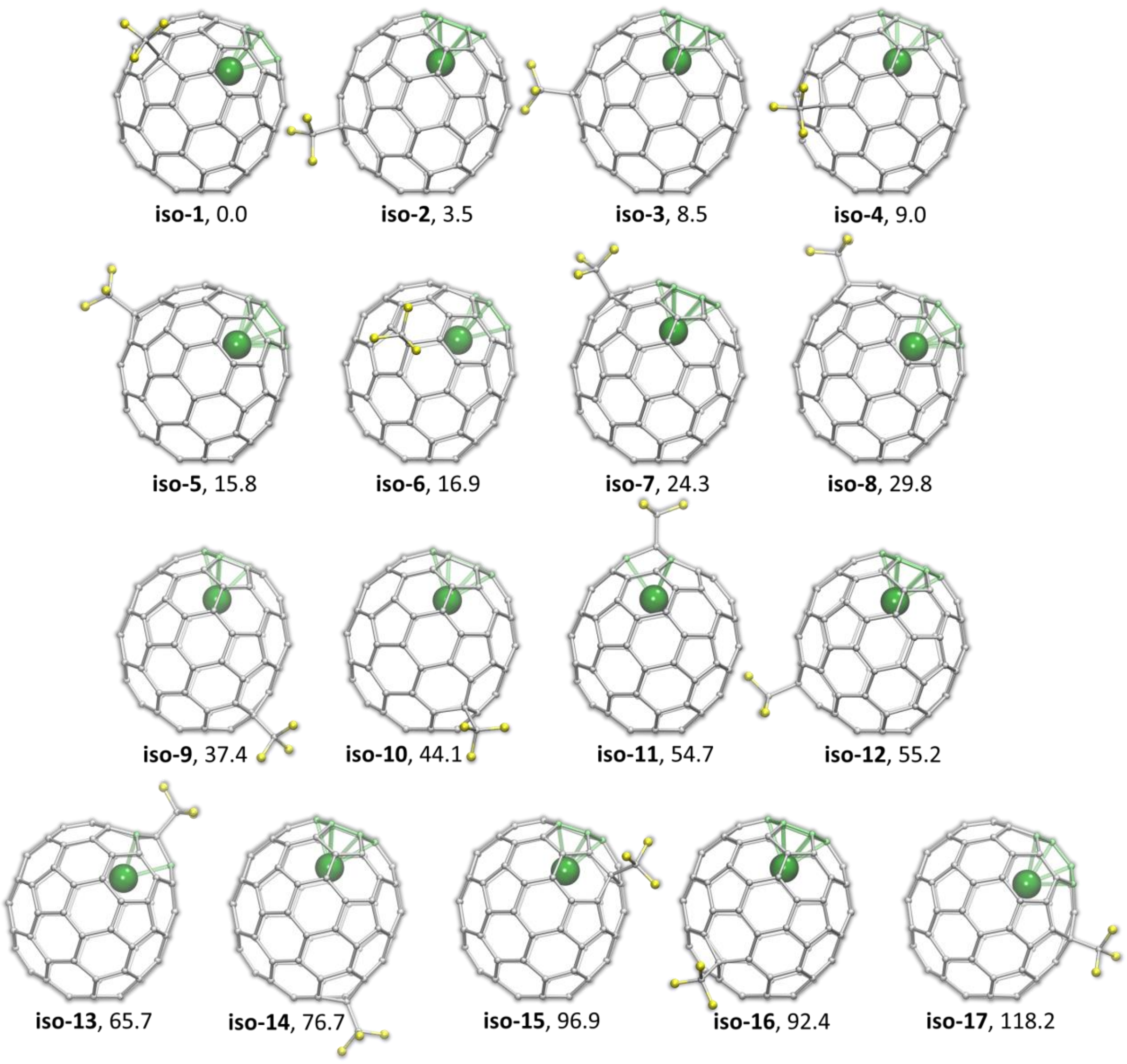

Figure S12. 17 regioisomers of Dy@C_{3v}(8)-$C_{82}(CF_3)$ and their relative energies (in kJ mol^{-1}) computed at the PBE0//PBE level. These relative energies were used in preparing the Figure 5a in the main text

Calculations were first performed for Y. Metal atom was placed in the center of the cage and allowed to migrate to the preferable position during optimization. In most cases optimized were positions close to that in the non-functionalized Dy@C_{3v}(8)-C_{82}. In few cases, when different metal positions were obtained, re-optimization with metal shifted to the position as in bare Dy@C_{3v}(8)-C_{82} showed lower energy. Thus, we can conclude one CF_3 groups does not strongly affect he preferable position of the metal. The Y@C_{3v}(8)-$C_{82}(CF_3)$ structures were then re-optimized at the PBE/def-TZVPP level with Dy (including Dolg's 4f-in-core ECP for Dy), and then single-point energies were calculated with PBE0 functional. Relative energies obtained with different functionals are listed in Table S4.

Table S4. Relative energies of 17 isomers of M@C_{3v}(8)-C_{82}(CF_3) (kJ mol^{-1})

	Y, PBE [a]	Dy, PBE [b]	Dy, PBE0//PBE [c]
**iso-1**	0.0	0.0	0.0
**iso-2**	0.5	−0.4	3.5
**iso-3**	5.3	4.6	8.5
**iso-4**	8.0	7.7	9.0
**iso-5**	11.0	11.4	15.8
**iso-6**	11.2	11.3	16.9
**iso-7**	12.7	16.7	24.3
**iso-8**	21.0	23.5	29.8
**iso-9**	28.2	27.4	37.8
**iso-10**	37.6	37.3	44.1
**iso-11**	43.9	49.3	54.7
**iso-12**	45.7	44.8	55.2
**iso-13**	49.8	56.1	65.7
**iso-14**	60.5	60.1	76.7
**iso-15**	68.9	75.4	96.9
**iso-16**	76.0	76.2	92.4
**iso-17**	93.6	99.7	118.2

[a] opt PBE – optimized at the PBE/TZ2P level with SBKJC-type ECP for Y, Priroda code;

[b] optimized at the PBE/def-TZVPP level with Dolg's 4f-in-core ECP for Dy, Orca code;

[c] Single-point calculations with PBE0 functional using geometry optimized at the PBE/def2-TZVPP level, basis set for Dy includes Dolg's 4f-in-core ECP, Orca code;

CASSCF calculations of LF splitting

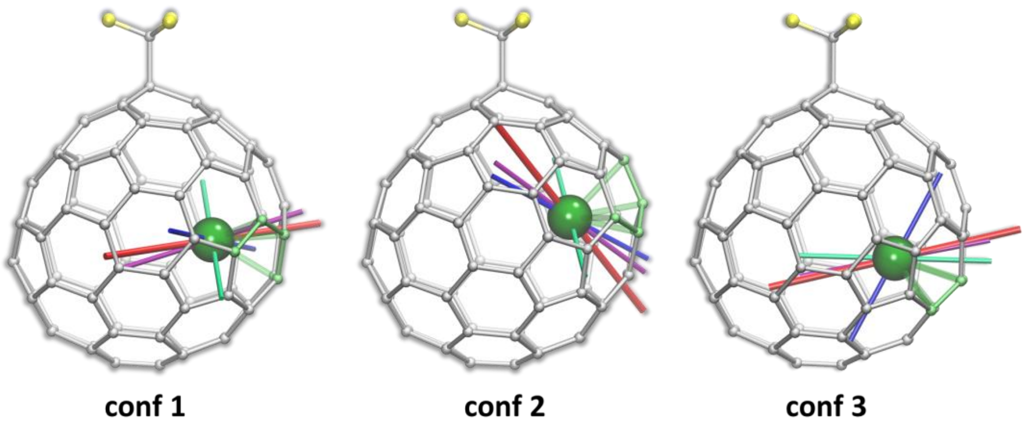

Figure S13. Orientations of principal g_z axes of four lowest Kramers doublets (KDs) in three conformers of Dy@C_{3v}(8)-$C_{82}(CF_3)$; principal g_z-axes are shown in red (KD1), cyan (KD2), magenta (KD3), and violet (KD4), axis of the ground-state doublet KD1 is highlighted by a larger size, carbon atoms with Dy–C distance shorter 2.5 Å are shown in light green. A perpendicular orientation of molecules is chosen versus the one shown in Figure 6 of the main text.

Table S5a. Dy@C_{3v}(8)-$C_{82}(CH_2Ph)$, **conf 1**. CASSCF-calculated LF splitting, g tensors of Kramers doublets (KDs), angle of g_z principal axes with respect to that of KD1, and weights in the basis of m_J states (in %)

	E	g_x	g_y	g_z	angle	|±15/2⟩	|±13/2⟩	|±11/2⟩	|±9/2⟩	|±7/2⟩	|±5/2⟩	|±3/2⟩	|±1/2⟩
KD1	0	1.38	2.79	15.30	0	**42**	9	**22**	**17**	2	4	3	0
KD2	35	1.19	3.67	13.24	33	14	**27**	7	**21**	**16**	7	6	2
KD3	67	1.27	5.03	9.72	20	9	13	**32**	7	**23**	12	2	3
KD4	104	8.74	6.40	0.67	60	9	10	2	10	**17**	**25**	**22**	6
KD5	135	0.76	3.74	9.84	55	11	**16**	1	8	8	8	12	**37**
KD6	165	0.71	1.53	16.14	73	2	6	1	12	12	**15**	**22**	**30**
KD7	194	4.67	5.10	9.55	66	11	**16**	**17**	5	5	**19**	**21**	8
KD8	243	0.37	0.47	16.30	68	3	3	**19**	**20**	**16**	11	13	**15**

Table S5b. Dy@C_{3v}(8)-$C_{82}(CF_3)$, **conf 1**. CASSCF-calculated LF splitting, g tensors of Kramers doublets (KDs), angle of g_z principal axes with respect to that of KD1 (°), and weights in the basis of m_J states (in %)

	E	g_x	g_y	g_z	angle	|±15/2⟩	|±13/2⟩	|±11/2⟩	|±9/2⟩	|±7/2⟩	|±5/2⟩	|±3/2⟩	|±1/2⟩
KD1	0	1.27	2.46	15.66	0	**47**	7	**19**	**19**	1	4	2	0
KD2	36	1.21	3.33	13.68	33	15	**26**	7	**21**	**18**	5	6	1
KD3	70	1.49	5.30	9.52	22	8	11	**33**	6	**28**	11	2	3
KD4	107	9.17	6.34	0.66	65	9	10	3	10	**16**	**30**	**18**	5
KD5	138	0.66	3.79	9.96	53	10	**19**	1	9	7	9	14	**33**
KD6	169	0.87	1.56	16.09	72	2	7	1	13	12	14	**23**	**31**
KD7	196	4.26	4.78	10.03	66	9	**18**	**17**	4	4	**17**	**22**	10
KD8	245	0.38	0.50	16.34	68	2	4	**20**	**19**	14	10	13	**18**

Table S6a. Dy@C_{3v}(8)-$C_{82}(CH_2Ph)$, **conf 2**. CASSCF-calculated LF splitting, *g* tensors of Kramers doublets (KDs), angle of g_z principal axes with respect to that of KD1, and weights in the basis of m_J states (in %)

	E	g_x	g_y	g_z	**angle**	**|±15/2⟩**	**|±13/2⟩**	**|±11/2⟩**	**|±9/2⟩**	**|±7/2⟩**	**|±5/2⟩**	**|±3/2⟩**	**|±1/2⟩**
KD1	0	3.13	4.91	13.04	0	5	**62**	9	1	5	7	2	8
KD2	32	1.73	5.40	9.62	29	13	13	**47**	4	1	4	8	8
KD3	60	1.18	2.39	11.56	25	**32**	14	5	**16**	9	3	14	7
KD4	94	0.85	6.04	11.49	67	9	4	9	14	11	13	**21**	**19**
KD5	122	1.33	3.59	6.21	81	13	3	8	**29**	11	13	8	**17**
KD6	145	0.61	1.63	15.52	86	8	1	6	6	14	26	16	24
KD7	183	2.20	3.80	9.70	65	**17**	1	**15**	**17**	**21**	3	14	14
KD8	265	0.20	0.27	17.65	69	4	2	2	13	**28**	**31**	**17**	4

Table S6b. Dy@C_{3v}(8)-$C_{82}(CF_3)$, **conf 2**. CASSCF-calculated LF splitting, *g* tensors of Kramers doublets (KDs), angle of g_z principal axes with respect to that of KD1 (°), and weights in the basis of m_J states (in %)

	E	g_x	g_y	g_z	**angle**	**|±15/2⟩**	**|±13/2⟩**	**|±11/2⟩**	**|±9/2⟩**	**|±7/2⟩**	**|±5/2⟩**	**|±3/2⟩**	**|±1/2⟩**
KD1	0	3.34	4.61	13.02	0	**28**	7	**40**	3	13	3	3	3
KD2	30	1.54	5.43	10.57	22	13	**34**	11	**20**	3	14	5	1
KD3	58	0.75	1.63	10.82	16	5	**47**	3	**23**	3	9	9	2
KD4	95	0.69	5.88	11.49	77	12	1	1	8	17	8	16	**37**
KD5	121	5.38	4.10	0.01	64	15	5	13	10	12	4	17	**25**
KD6	141	1.05	1.73	15.89	85	5	3	3	5	14	**25**	**34**	10
KD7	181	2.73	4.77	9.38	59	**18**	4	**21**	10	10	**16**	4	**18**
KD8	260	0.25	0.32	17.61	65	4	0	8	**22**	**27**	**22**	13	4

Table S7a. Dy@C_{3v}(8)-$C_{82}(CH_2Ph)$, **conf 3**. CASSCF-calculated LF splitting, *g* tensors of Kramers doublets (KDs), angle of g_z principal axes with respect to that of KD1, and weights in the basis of m_J states (in %)

	E	g_x	g_y	g_z	**angle**	**|±15/2⟩**	**|±13/2⟩**	**|±11/2⟩**	**|±9/2⟩**	**|±7/2⟩**	**|±5/2⟩**	**|±3/2⟩**	**|±1/2⟩**
KD1	0	0.24	0.38	18.94	0	**91**	0	0	4	2	2	0	0
KD2	55	1.97	4.64	14.19	48	5	1	3	**33**	**48**	5	2	2
KD3	87	0.03	3.66	8.82	4	1	0	**19**	**33**	**25**	**17**	4	1
KD4	132	2.27	4.97	9.02	23	2	11	**15**	0	11	**56**	2	2
KD5	153	0.21	0.63	9.05	5	0	**28**	**19**	12	4	**18**	14	5
KD6	173	0.29	1.15	12.53	66	0	4	14	5	7	1	**51**	**18**
KD7	223	7.41	7.22	5.01	34	0	**31**	0	3	0	1	**21**	**43**
KD8	277	0.88	1.44	16.27	53	0	**25**	**28**	9	2	0	5	**30**

Table S7b. Dy@C_{3v}(8)-$C_{82}(CF_3)$, **conf 3**. CASSCF-calculated LF splitting, *g* tensors of Kramers doublets (KDs), angle of g_z principal axes with respect to that of KD1 (°), and weights in the basis of m_J states (in %)

	E	g_x	g_y	g_z	**angle**	**|±15/2⟩**	**|±13/2⟩**	**|±11/2⟩**	**|±9/2⟩**	**|±7/2⟩**	**|±5/2⟩**	**|±3/2⟩**	**|±1/2⟩**
KD1	0	0.13	0.24	19.22	0	**94**	0	0	3	1	2	0	0
KD2	66	1.84	4.89	14.09	49	4	0	3	**36**	**49**	5	2	2
KD3	97	0.50	3.49	8.86	2	1	0	**19**	**34**	**26**	**16**	4	1
KD4	142	2.43	4.16	9.25	15	2	14	**19**	0	9	**52**	2	2
KD5	161	0.97	1.18	9.12	3	0	**27**	**17**	12	6	**23**	11	4
KD6	185	0.28	1.01	12.89	63	0	5	**16**	5	6	1	**51**	15
KD7	233	7.20	7.11	5.36	39	0	**32**	1	3	0	1	**24**	**40**
KD8	285	1.07	1.84	15.91	57	0	**21**	**26**	8	2	1	6	**36**

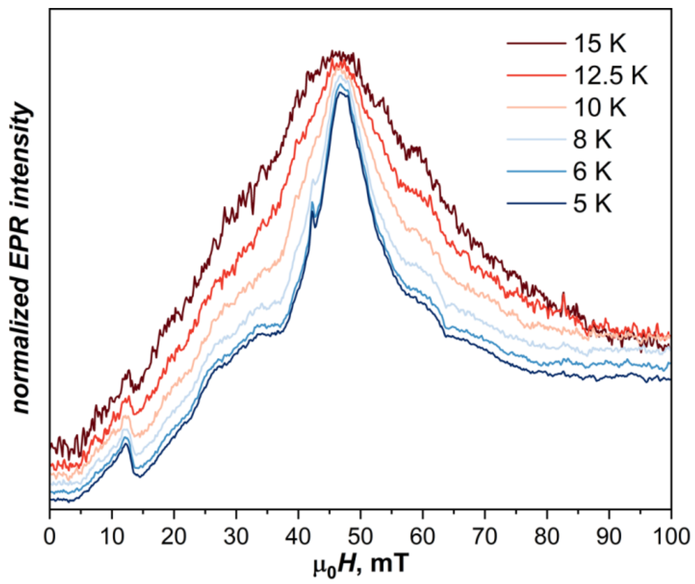

Figure S14. Normalized X-band EPR spectra of Dy@C_{3v}(8)-C_{82}(CH_2Ph) measured at different temperatures in glassy frozen solution in *o*-terphenyl. Broadening of the signal with temperature can be clearly seen in this representation.

Supplementary References

1. Liu, F.; Krylov, D. S.; Spree, L.; Avdoshenko, S. M.; Samoylova, N. A.; Rosenkranz, M.; Kostanyan, A.; Greber, T.; Wolter, A. U. B.; Büchner, B.; et al. Single molecule magnet with an unpaired electron trapped between two lanthanide ions inside a fullerene. *Nat. Commun.* **2017**, *8*, 16098.
2. Wang, Y.; Velkos, G.; Israel, N. J.; Rosenkranz, M.; Büchner, B.; Liu, F.; Popov, A. A. Electrophilic Trifluoromethylation of Dimetallofullerene Anions en Route to Air-Stable Single-Molecule Magnets with High Blocking Temperature of Magnetization. *J. Am. Chem. Soc.* **2021**, *143* (43), 18139-18149.
3. Mueller, U.; Förster, R.; Hellmig, M.; Huschmann, F. U.; Kastner, A.; Malecki, P.; Pühringer, S.; Röwer, M.; Sparta, K.; Steffien, M.; et al. The macromolecular crystallography beamlines at BESSY II of the Helmholtz-Zentrum Berlin: Current status and perspectives. *Eur. Phys. J. Plus* **2015**, *130* (7), 141.
4. Kabsch, W. XDS. *Acta Cryst. D* **2010**, *66* (2), 125-132.
5. Sparta, K. M.; Krug, M.; Heinemann, U.; Mueller, U.; Weiss, M. S. XDSAPP2.0. *J. Appl. Crystallogr.* **2016**, *49* (3), 1085-1092.
6. Sheldrick, G. Crystal structure refinement with SHELXL. *Acta Cryst. C* **2015**, *71* (1), 3-8.
7. Chilton, N. F.; Anderson, R. P.; Turner, L. D.; Soncini, A.; Murray, K. S. PHI: A powerful new program for the analysis of anisotropic monomeric and exchange-coupled polynuclear d- and f-block complexes. *J. Comput. Chem.* **2013**, *34* (13), 1164-1175.
8. Perdew, J. P.; Burke, K.; Ernzerhof, M. Generalized gradient approximation made simple. *Phys. Rev. Lett.* **1996**, *77* (18), 3865-3868.
9. Laikov, D. N.; Ustynuk, Y. A. PRIRODA-04: a quantum-chemical program suite. New possibilities in the study of molecular systems with the application of parallel computing. *Russ. Chem. Bull.* **2005**, *54* (3), 820-826.
10. Laikov, D. N. Fast evaluation of density functional exchange-correlation terms using the expansion of the electron density in auxiliary basis sets. *Chem. Phys. Lett.* **1997**, *281*, 151-156.
11. Ask Hjorth, L.; Jens Jørgen, M.; Jakob, B.; Ivano, E. C.; Rune, C.; Marcin, D.; Jesper, F.; Michael, N. G.; Bjørk, H.; Cory, H.; et al. The atomic simulation environment—a Python library for working with atoms. *J. Phys. Condens. Matter* **2017**, *29* (27), 273002.
12. Neese, F.; Wennmohs, F.; Becker, U.; Riplinger, C. The ORCA quantum chemistry program package. *J. Chem. Phys.* **2020**, *152* (22), 224108.
13. Neese, F. Software update: the ORCA program system, version 4.0. *WIREs Comput. Mol. Sci.* **2018**, *8* (1), e1327.
14. Dolg, M.; Stoll, H.; Savin, A.; Preuss, H. Energy-adjusted pseudopotentials for the rare earth elements. *Theor. Chim. Acta* **1989**, *75* (3), 173-194.
15. Yang, J.; Dolg, M. Valence basis sets for lanthanide 4f-in-core pseudopotentials adapted for crystal orbital ab initio calculations. *Theor. Chem. Acc.* **2005**, *113* (4), 212-224.
16. Weigend, F.; Ahlrichs, R. Balanced basis sets of split valence, triple zeta valence and quadruple zeta valence quality for H to Rn: Design and assessment of accuracy. *Phys. Chem. Chem. Phys.* **2005**, *7*, 3297-3305.
17. Hafner, J. Ab-initio simulations of materials using VASP: Density-functional theory and beyond. *J. Comput. Chem.* **2008**, *29* (13), 2044-2078.
18. Kresse, G.; Hafner, J. Ab initio molecular dynamics for liquid metals. *Phys. Rev. B* **1993**, *47* (1), 558-561.

19. Kresse, G.; Joubert, D. From ultrasoft pseudopotentials to the projector augmented-wave method. *Phys. Rev. B* **1999**, *59* (3), 1758-1775.
20. Grimme, S. Density functional theory with London dispersion corrections. *WIREs Comput. Mol. Sci.* **2011**, *1* (2), 211-228.
21. Aquilante, F.; Autschbach, J.; Baiardi, A.; Battaglia, S.; Borin, V. A.; Chibotaru, L. F.; Conti, I.; Vico, L. D.; Delcey, M.; Galván, I. F.; et al. Modern quantum chemistry with [Open]Molcas. *J. Chem. Phys.* **2020**, *152* (21), 214117.
22. Chibotaru, L. F.; Ungur, L. Ab initio calculation of anisotropic magnetic properties of complexes. I. Unique definition of pseudospin Hamiltonians and their derivation. *J. Chem. Phys.* **2012**, *137* (6), 064112.
23. Humphrey, W.; Dalke, A.; Schulten, K. VMD - Visual Molecular Dynamics. *J. Molec. Graphics* **1996**, *14*, 33-38.
